# Transcriptional and Post-Translational Regulation of Junctional Adhesion Molecule-B (JAM-B) in Leukocytes under Inflammatory Stimuli

**DOI:** 10.3390/ijms23158646

**Published:** 2022-08-03

**Authors:** Priscilla E. Day-Walsh, Bryony Keeble, Gothai Pirabagar, Samuel J. Fountain, Paul A. Kroon

**Affiliations:** 1 Quadram Institute Bioscience, Food Innovation & Health Programme, Norwich Research Park, Rosalind Franklin Road, Norwich NR4 7UQ, UK; priscilla.day-walsh@quadram.ac.uk (P.E.D.-W.); bryonykeeble97@gmail.com (B.K.); gothai.pirabagar@gmail.com (G.P.); 2 School of Biological Sciences, University of East Anglia, Norwich Research Park, Norwich NR4 7TJ, UK; s.j.fountain@uea.ac.uk

**Keywords:** pathogen, cell adhesion, cell migration, cell permeability, host, inflammation, tight junctions, barrier function

## Abstract

Junctional adhesion molecules (JAMs; comprising JAM-A, -B and -C) act as receptors for viruses, mediate cell permeability, facilitate leukocyte migration during sterile and non-sterile inflammation and are important for the maintenance of epithelial barrier integrity. As such, they are implicated in the development of both communicable and non-communicable chronic diseases. Here, we investigated the expression and regulation of JAM-B in leukocytes under pathogen- and host-derived inflammatory stimuli using immunoassays, qPCR and pharmacological inhibitors of inflammatory signalling pathways. We show that JAM-B is expressed at both the mRNA and protein level in leukocytes. JAM-B protein is localised to the cytoplasm, Golgi apparatus and in the nucleus around ring-shaped structures. We also provide evidence that JAM-B nuclear localisation occurs via the classical importin-α/β pathway, which is likely mediated through JAM-B protein nuclear localisation signals (NLS) and export signals (NES). In addition, we provide evidence that under both pathogen- and host-derived inflammatory stimuli, JAM-B transcription is regulated via the NF-κB-dependent pathways, whereas at the post-translational level JAM-B is regulated by ubiquitin-proteosome pathways. Anaphase-promoting ubiquitin ligase complex (APC/C) and herpes simplex virus-associated ubiquitin-specific protease (HAUSP/USP) were identified as candidates for JAM-B ubiquitination and de-ubiquitination, respectively. The expression and regulation of JAM-B in leukocytes reported here is a novel observation and contrasts with previous reports. The data reported here suggest that JAM-B expression in leukocytes is under the control of common inflammatory pathways.

## 1. Introduction

Junctional adhesion molecules (JAMs) play a key role in in the pathogenesis of both communicable and non-communicable diseases by acting as receptors for viruses and mediating a broad range of cellular processes, including haematopoiesis, chemokine secretion, cell migration and cell adhesion [1,2,3,4,5,6,7]. Due to their role in tight junctions, they are also critical to the maintenance of lung, gastrointestinal and blood–brain barrier integrity [8,9].

Three major types of JAMs have been identified (JAM-A, JAM-B and JAM-C, also known as JAM-A/F11R or JAM-1, JAM-B/VE-JAM or JAM-2, and JAM-C or JAM-3, respectively) [5,10,11,12,13,14,15,16]. Other JAM-like proteins have also been described [17,18]. While there are numerous reports of the functions of JAM-A and JAM-C in leukocytes, the role of JAM-B is less clear and remains a matter of debate [2,12,15,19,20,21,22]. Based on the Uniprot database, there are three JAM-B isoforms, isoform 1 (33.2 kDa, 298 amino acids), isoform 2 (29 kDa, missing amino acids in positions 44–79) and isoform 3 (34.6 kDa, 312 amino acids) [23]. To be functional, JAMs must interact with each other or with other adhesion molecules such as integrins in a *cis* or *trans* configuration [6]. All JAMs can form homotypic interactions to form homodimers, but these become dissociated upon heterotypic interactions (i.e., with other proteins) [6]. JAM-B interacts with an integrin dimer α4β1, also called very late antigen-4 (VLA4), but the interaction has been shown to require prior interaction of JAM-B with JAM-C, and therefore, this likely only occurs in cells that co-express JAM-C and VLA4 [24].

While expression of JAM-A and JAM-C has been reported in leukocytes and platelets, the existing literature suggests that expression of JAM-B is confined to endothelial cell-cell junctions [10,14,22]. Arrate et al. demonstrated that JAM-C, although it can form heterophilic interaction with JAM-B in ovarian cells, was not able to bind to leukocyte cells in static adhesion assays [10], while Cunningham et al. reported negligible binding of JAM-B to B-lymphocytes and monocytic cell lines including THP-1 monocytes [23]. The authors of both these reports suggested that this indicated a lack of JAM-B expression in leukocytes [10,24]. The interaction of JAM-C and VLA-4 present on leukocytes/lymphocytes with JAM-B on endothelial cells has been suggested as a mechanism through which JAM-C, JAM-B and VLA-4 modulate inflammatory processes [10,14]. Nevertheless, others have speculated that the ability of JAM-B to adhere to other adhesion molecules may be determined by inflammatory activation, as observed in contact hypersensitivity [25]. Thus, the inability for JAM-C and JAM-B to bind to leukocytes may not necessarily indicate a lack of JAM-B expression. Because JAM-B plays a critical role in lymphocyte homing, leukocyte migration and adhesion, and haematopoiesis, we sought to provide clarity on the expression and regulation of JAM-B in leukocytes [19,20,21].

Here, we used quantitative PCR, immunofluorescence and fluorescent microscopy, and Western blotting to characterise the expression and localisation of JAM-B in leucocytes at the gene and protein level. We determined JAM-B responses to pathogen- and host-derived inflammatory stimuli and identified the signalling pathways involved in regulating JAM-B expression. Using computational biology approaches, we further identified JAM-B nuclear export signals and JAM-B interacting partners that may influence its localisation, regulation and function.

## 2. Results

### 2.1. JAM-B Is Expressed in THP-1 Cells, Human PBMCs and Macrophages at mRNA Level

**Figure 1:**
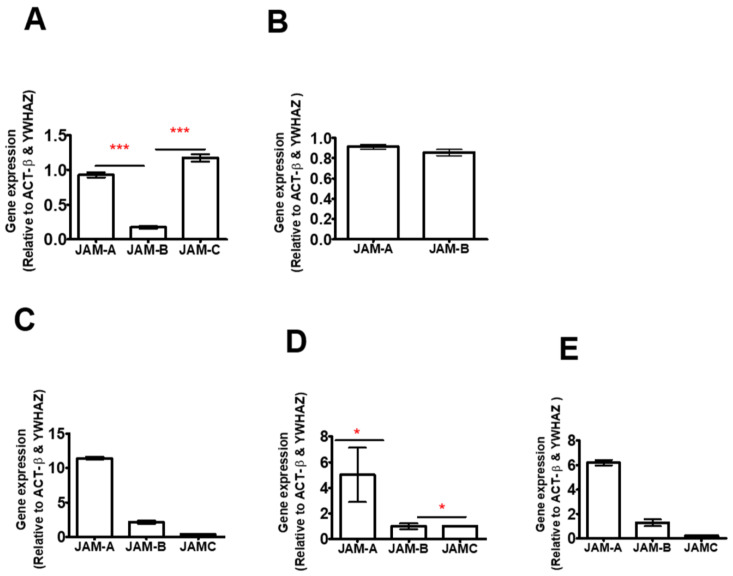
Relative gene expression of JAMs in (**A**) THP-1 monocytes, (**B**) PMA-differentiated THP-1 macrophages, (**C**) primary human monocytes, (**D**) differentiated primary macrophages and (**E**) peripheral blood mononuclear cells (PBMC). * indicates *p ≤* 0.05 *** indicates *p* ≤ 0.0001 in comparison to each other. *n* = 3 for cell lines and *n* = 1 donor with 3 replicates for primary cells as such statistics not carried out for primary cells. Data are mean ± SEM.

Gene expression of JAM-A, JAM-B and JAM-C was first determined in unstimulated THP-1 monocytes (cancer-derived cell line) and PMA-differentiated THP-1 macrophages by real-time qPCR using validated primers (Table 1 and Supplementary Figure S1B,C). All JAMs were found to be expressed in THP-1 monocytes (Figure 1A), while only JAM-A and JAM-B were expressed in THP-1-differentiated macrophages (Figure 1B). The PCR product from the JAM-B PCR assay was fully sequenced, confirming that the product was from the JAM-B transcripts (Supplementary Figure S2). There were differences in the relative expression of each JAM in THP-1 monocytes (χ^2^ (2) = 79.84), with JAM-B gene expression being 5-fold lower than that of JAM-A (*p* ≤ 0.001), but that of JAM-A was not different from JAM-C (*p* ≥ 0.05) (Figure 1A and Supplementary Table S1). In THP-1 monocytes, the relative expression of JAM-B was also 7-fold lower than that of JAM-C (*p* ≤ 0.001) (Figure 1A and Supplementary Table S1). In contrast to THP-1 monocytes, JAM-C was not expressed in PMA-differentiated THP macrophages, while the relative expression of JAM-B was not different from that of JAM-A (U = 569, *p* = 0.21) (Figure 1B and Supplementary Table S1).

The gene expression of the JAMs was further assessed in human primary macrophages (*n* = 4 donors) and in primary monocytes and peripheral mononuclear cells (PBMCs) from one donor in three replicates. All JAMs were expressed in primary leukocytes and in PBMCs (Figure 1 and Supplementary Table S1). The relative expression of JAM-B in CD14+ monocytes was 5-fold lower and 6-fold higher than that of JAM-A and JAM-C, respectively, while that of JAM-A was 30-fold higher than the expression of JAM-C (Figure 1C and Supplementary Table S1). In primary granulocyte–macrophage colony-stimulating factor (GM-CSF)-differentiated macrophages, the relative expression of JAM-B and JAM-C was 5-fold lower than that of JAM-A (Figure 1C and Supplementary Table S1). However, there were no differences in the relative expression of JAM-B and JAM-C in GM-CSF macrophages (Figure 1D and Supplementary Table S1). This contrasts with THP-1 monocytes (Figure 1A), where the relative expression of JAM-B was lower than that of JAM-C. Unlike in THP-1-differentiated macrophages, JAM-C was expressed in primary macrophages, albeit at low levels (Figure 1D). In PBMCs, the relative expression of JAM-B was lower than that of JAM-A but higher than that of JAM-C, and the relative expression of JAM-B was slightly higher than that of JAM-C (Figure 1E and Supplementary Table S1). JAM-C expression was 29-fold lower than that of JAM-A in PBMCs (Figure 1E).

### 2.2. TNF-α and LPS Up-Regulate JAM-B Gene Expression in THP-1 Monocytes and PMA Differentiated THP-1 Macrophages

**Figure 2:**
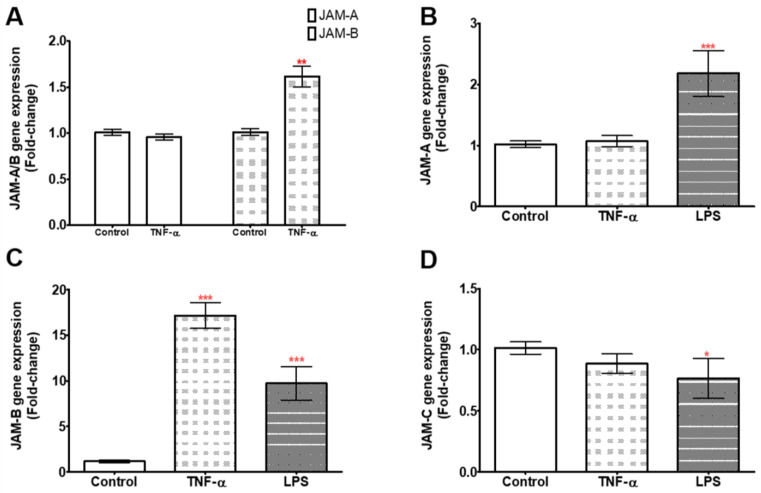
Fold-changes in JAM gene expression in response to TNF-α and LPS treatments for 24 h relative to the geometric mean of YWHAZ and ACT-β expression. All data are the relative expression of the JAMs treated with TNF-α or LPS compared to the respective non-treated control. (**A**) JAM-A and JAM-B expression in THP-1-differentiated macrophages. (**B**–**D**) Relative expression of JAM-A, JAM-B and JAM-C, respectively, in THP-1 monocytes. *** indicates *p* ≤ 0.0001, ** indicates *p* ≤ 0.01, * indicates *p* ≤ 0.05. *n* = 9 for TNF-α treatments and *n* = 6 for LPS treatments. Data are mean ± SEM.

We further determined whether the expression of JAMs, particularly JAM-B, was regulated by microbial (1 µg/mL LPS) and host (10 ng/mL TNF-α) inflammatory stimuli by treating the cells for 24 h with LPS or TNF-α. In THP-1-differentiated macrophages, no differences in JAM-A gene expression were observed between the control and TNF-α-treated samples (U = 11, *p* = 0.310, *n* = 6) (Figure 2A and Supplementary Table S2), while JAM-B gene expression was significantly up-regulated by approximately 1.6-fold in the presence of TNF-α (U = 0, *p* = 0.002) (Figure 2A and Supplementary Table S2). In THP-1 monocytes, TNF-α had no effect on the expression of JAM-A (U = 30, *p* = 0.386, *n* = 9) or JAM-C (U = 23, *p* = 0.136, *n* = 9) (Figure 2B,D and Supplementary Table S2), whereas the expression of JAM-B was increased by 17-fold (U = 0, *p* = 0.0001, *n* = 9) (Figure 2C and Supplementary Table S2). The response of JAM-B to TNF-α in THP-1 monocytes was 11-fold higher than that observed in PMA-differentiated THP-1 macrophages, whereas the JAM-A response was comparable between the two cell types. We next investigated JAM expression following treatment with LPS (1 µg/mL) in THP-1 monocytes. JAM-A and JAM-B expression significantly increased by ~2-fold (U = 1.0, *p* = 0003, *n* = 6) (Figure 2B and Supplementary Table S2) and ~10-fold (U = 0.0, *p* = 0.0001, *n* = 6), respectively (Figure 2C and Supplementary Table S2). In contrast, the expression of JAM-C was down-regulated by LPS by 0.8-fold (U = 11.0, *p* = 0.031, *n* = 6) (Figure 2D and Supplementary Table S2). However, there was no change in the expression of JAM-C in response to TNF-α.

**Figure 3:**
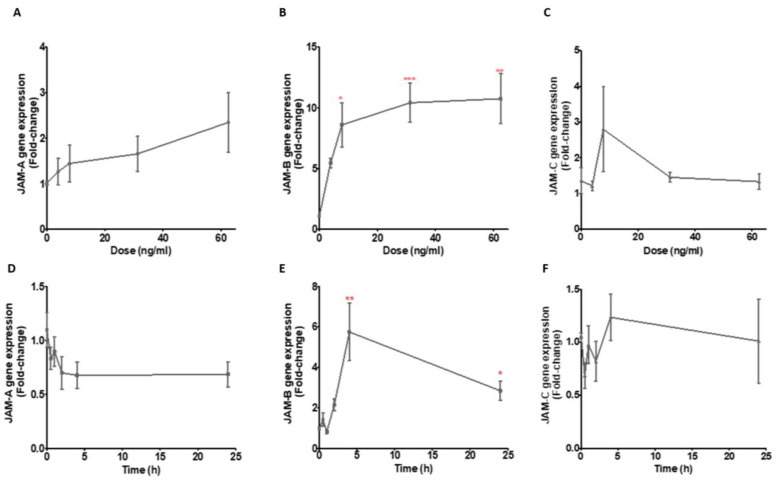
Gene expression responses of the JAMs to varying LPS doses relative to the geometric mean of YWHAZ and ACT-β expression (**A**–**C**) and to 10 ng/mL LPS over a 24 h time course with sampling at 0, 0.5, 1, 2 and 4 h and then at 24 h in THP-1 monocytes (**D**–**F**). (**A**,**D**) JAM-A gene expression, (**B**,**E**) JAM-B gene expression and (**C**,**F**) JAM-C gene expression. The comparisons in (**A**) to (**C**) are between the control without LPS and the different LPS doses while those in (**D**) to (**C**) are between expression at time 0 and at various times. *** indicates *p* ≤ 0.0001, ** indicates *p* ≤ 0.001, * indicates *p* ≤ 0.05.

Having demonstrated changes in the gene expression of the JAMs in response to treatment with a high concentration of LPS, we next investigated the responses of the JAMs to physiological LPS concentrations ranging from 3.9 ng/mL to 62.5 ng/mL, again following a 24 h incubation. Although the relative abundance of transcripts for JAM-A increased with increasing concentrations of LPS to >2-fold at the highest concentration of LPS, the differences were not significant (no statistical differences in JAM-A gene expression comparing 3.6 to 62.5 ng/mL concentrations in comparison to controls (χ^2^ (2) = 8.62, *p* = 0.071, *n* = 6) (Figure 3A and Supplementary Table S3). In contrast, the relative expression of JAM-B was increased up to ~10-fold at the highest concentration tested compared to the control, with significant increases in expression at 7.8, 31.25 and 62.5 ng/mL LPS (χ^2^ (2) = 19.56, *p* = 0.0006, *n* = 6) (Figure 3B and Supplementary Table S3). There were no differences in the expression of JAM-C at all LPS doses in comparison to the control (χ^2^ (2) = 5.3, *p* = 0.26, *n* = 6) (Figure 3C and Supplementary Table S3).

Next, we assessed changes in JAM expression over a 4 h time course and at 24 h in response to treatment with a physiological concentration (10 ng/mL) of LPS. The relative expression (compared to the zero-time point) of JAM-A appeared to decrease over the first 2 h and remained at this reduced level up to 24 h, but this change was not statistically different from the control (χ^2^ (2) = 5.9, *p* = 0.32, *n* = 5) (Figure 3D and Supplementary Table S3). The expression of JAM-B started to increase at 2 h, was highest at 4 h (χ^2^ (2) = 22.15, *p* = 0.0005, *n* = 5) and the expression at 24 h was modestly reduced from the level at 4 h (Figure 3E and Supplementary Table S3). There were no significant changes in the expression of JAM-C over time after treatment with 10 ng/mL LPS (χ^2^ (2) = 4.54, *p* = 0.47, *n* = 2) (Figure 3F and Supplementary Table S3). It is noteworthy that there is a mismatch between the data for the dose response and the time responses for both JAM-A (Figure 3A,D) and JAM-B (Figure 3B,E) despite the cells being treated at similar passage numbers. It is possible that media and FBS batch variations might have led to the dampening of the response, as observed in a previous study by Yang et al. [26].

### 2.3. JAM-B Gene Expression Is Regulated by Nuclear Factor-κB (NF-κB)

To investigate the mechanisms regulating JAM-B gene expression, we used pharmacological inhibitors of the NF-κB regulatory pathways, Bay 11-7082 and p38 MAP Kinase Inhibitor IV (MT4), respectively. The inhibitors were added to control cells (without inflammatory stimuli/unstimulated) or to TNF-α- and LPS-treated cells at concentrations of 10 µM and 130 nM, previously shown to inhibit the NF-κB and p38α/β MAPK regulatory pathways, respectively [27,28,29]. Comparisons were made between controls and controls plus inhibitor, LPS and LPS plus inhibitor, and TNF-α and TNF-α plus inhibitor.

**Figure 4:**
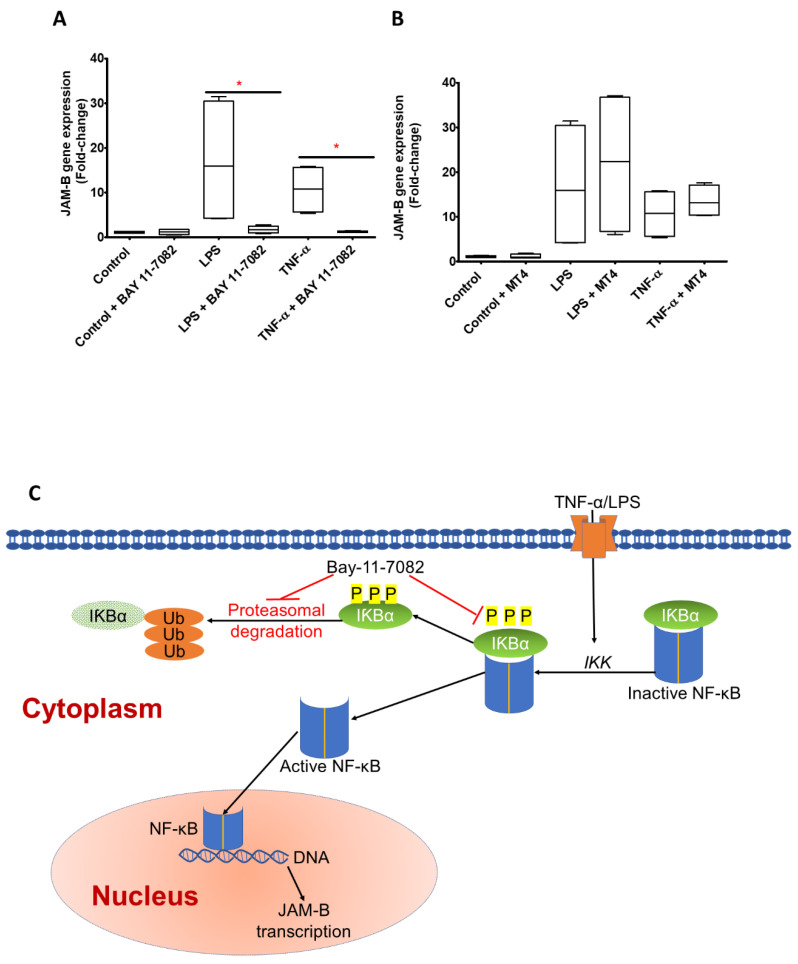
Regulation of JAM-B gene expression relative to the geometric mean of YWHAZ and ACT-β expression. (**A**) In THP1-1 monocytes, treatment with 10 µM of the NF-κB inhibitor Bay-11-7082 for 24 h did not further reduce JAM-B gene expression in the control samples but reduced JAM-B gene expression in the LPS (10 ng/mL) and TNF-α (10 ng/mL) treated samples (*p* = 0.029, *n* = 4). (**B**) Addition of the p38α/β MAPK inhibitor (MT4) had no effect on JAM-B gene expression in control, LPS or TNF-α incubations. (**C**) Mechanisms through which the Bay-11-7082 inhibitor prevents TNF-α or LPS induced JAM-B gene expression mediated by NF-κβ. Bay-11-7082 inhibits the phosphorylation of IKβ that is bound to inactive NF-κβ and the formation of poly-ubiquitination chains that aid the degradation of IKβ, i.e., the effect of Bay-11-7082 is to prevent the phosphorylation and ubiquitin-dependent proteasomal degradation of IKβ which remains bound to NF-κB and effectively blocks the NF-κβ nuclear localisation sequence so that NF-κβ does not translocate to the nucleus and become active to induce JAM-B transcription. The observation that treatment with Bay-11-7082 completely abolishes the TNFα- and LPS-induced increases JAM-B gene expression strongly suggests that the TNFα- and LPS-induced increases in JAM-B expression are a consequence of increased phosphorylation and proteasomal degradation of IKβ. * indicates *p* ≤ 0.05.

There were no statistical differences between the control (Mdn = 1.096, 0.87–1.29, *n* = 4) and the Bay 11-7082-only control-treated cells (Mdn = 1.12, 1.75–1.78), (U = 8.0, *p* = 1, *n* = 4) (Figure 4A). However, there were substantial and statistically significant reductions in JAM-B gene expression in response to Bay 11-7082 in the cells also treated with LPS (LPS only, Mdn = 15.9, 4.20–30.43; LPS plus Bay 11-7082, Mdn = 1.71, 0.94–2.51), (U = 0.0, *p* = 0.029, *n* = 4) and in cells also treated with TNF-α (TNF-α only, Mdn = 10.80, 5.62–15.62; TNF-α plus Bay 11-7082, Mdn = 1.16, 1.04–1.45), (U = 0.0, *p* = 0.029, *n* = 4) (Figure 4A). The Bay 11-7082 inhibitor completely reverted the substantial increases in JAM-B gene expression observed in response to treatments with LPS or TNF-α to similar levels as the control. The p38 MAP kinase inhibitor had no significant effect on unstimulated control cells or the LPS- or TNF-α-treated cells (Figure 4B), providing strong evidence that this signalling pathway is not involved in the TNF-α- and LPS-induced increases in JAM-B gene expression. Based on these results, we propose that the increases in expression of JAM-B caused by treatment of monocytes with LPS or TNF-α involves the activation of NF-κB. The activation occurs as a result of IκBα kinase (IKK)-mediated phosphorylation and ubiquitination of the inhibitor of κBα (IκBα), which leads to dissociation of IκBα from NF-κB, which activates NF-κB and allows it to translocate to the nucleus, where it binds specific response elements and activates the expression of the JAM-B gene (Figure 4C) [30].

### 2.4. JAM-B Has Polarised Localisation at the Cell Surface and Is Localised to the Cis-Golgi Network as Well as the Cytoplasm and the Nucleus

Using immunocytochemistry, we examined the expression of JAM-A and JAM-B proteins in monocytes and macrophages. Unlike JAM-A (Supplementary Figure S3A,B), which was strongly expressed at the cell surface and cell–cell junctions, JAM-B showed a strong intracellular punctate polarised expression (and, in some cases, granular cytoplasmic expression) (Supplementary Figure S3C,D). The strong JAM-B staining in monocytes was observed in structures resembling the Golgi apparatus. JAM-B staining was weak in the cytoplasm and at the cell surface.

**Figure 5:**
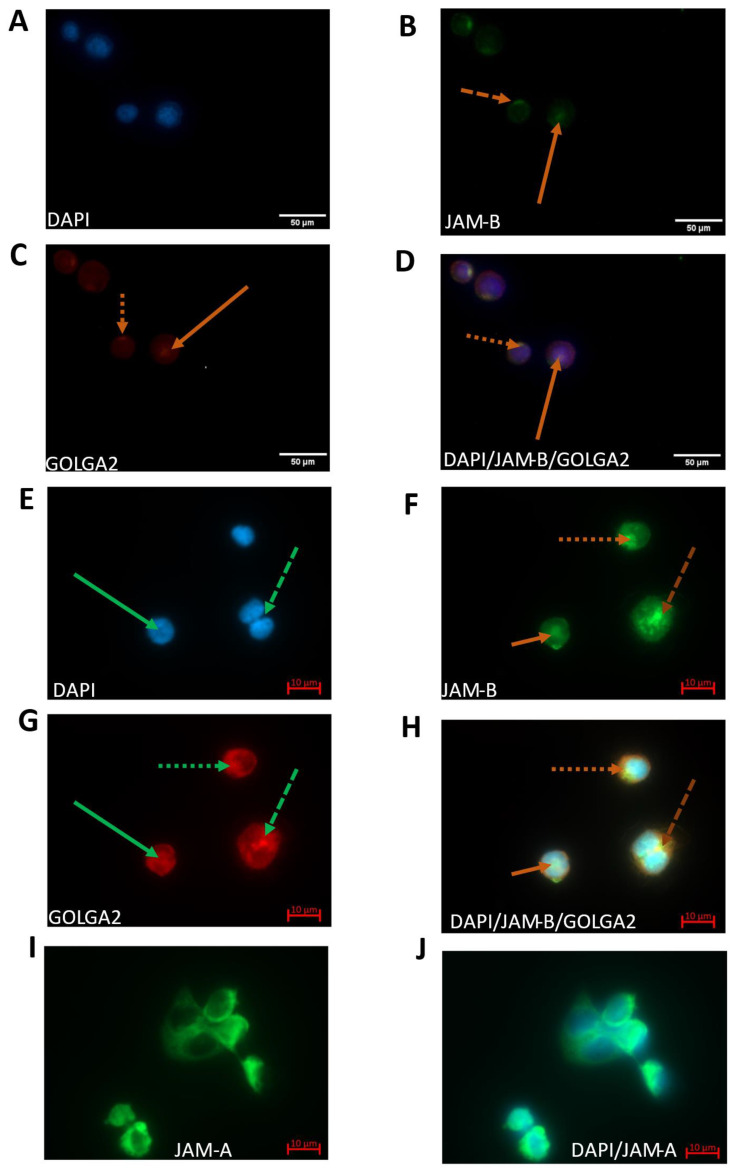
Immunofluorescence of DAPI, JAM-B and G130 using a mouse anti-JAM-B monoclonal antibody (SC-293496) and an anti-GM130 polyclonal antibody (PA1-077) in THP-1 monocytes (**A**–**D**) and macrophages (**E**–**J**). (**A**) DAPI nuclear staining in THP-1 monocytes; (**B**) JAM-B staining in THP-1 monocytes; (**C**) G130 Golgi staining in THP-1 monocytes (stains peripheral membrane component of the cis-Golgi stack marker GOLGA2); (**D**) composite image showing co-localisation of JAM-B and G130; (**E**) DAPI staining in THP-1-differentiated macrophages; (**F**) JAM-B staining in THP-1-differentiated macrophages showing polarised staining (dotted line), in splitting nuclei (dashed line), inside the nucleus (solid line); (**G**) cis-Golgi stack marker GOLGA2; (**H**) composite image showing DAPI, JAM-B and GOLGA2 staining. At least three technical replicates were carried out. JAM-B staining contrasts with that of JAM-A (**I**) (JAM-A staining) and (**J**) (composite JAM-A and DAPI staining).

Immunostaining was used in THP-1 monocytes to further confirm the localisation of JAM-B to the Golgi apparatus using the peripheral membrane component of the cis-Golgi stack marker GOLGA2 (GM130). A mouse anti-JAM-B monoclonal antibody was used instead of the anti-rabbit antibody used in the immunoblot analyses to confirm that the staining pattern observed was not due to antibody non-specificity. THP-1 monocytes and macrophages were counter-stained with DAPI, GOLGA2 and JAM-B (Figure 5). There was strong punctate polarised staining of JAM-B at the cell surface (Figure 5B). A similar staining pattern was also observed with GOLGA2 in THP-1 monocytes (Figure 5C), indicating co-localisation of JAM-B with GOLGA2 in the cis-Golgi stack (Figure 5D). Surprisingly, in a subset of THP-1 monocytes, co-staining of JAM-B and GOLGA2 was also observed in the nucleus (Figure 5A–C). The expression of JAM-B was further investigated in THP-1-differentiated macrophages. As well as polarised localisation to the Golgi (dotted lines), JAM-B staining was also observed inside the nucleus (solid lines) resembling nuclei in prophase (dashed lines) in between dividing nuclei resembling nuclei in anaphase [31,32] with somewhat weaker granular staining also in the cytoplasm. Interestingly, the JAM-B nuclear staining was consistently similar to that observed by Lordier et al. for the male germ cell Rac GTPase-activating protein (MgcRacGAP) in cells undergoing mitotic and endomitotic division, indicating possible involvement in cell cycle (Supplementary Figure S4C–E) [33]. Cytoplasmic staining was further demonstrated using Phalloidin, JAM-B and DAPI staining (Supplementary Figure S4F,G). However, as we did not investigate JAM-B expression in relation to cell cycle progression, further work is required to validate these observations. GOLGA2 contains a nuclear localisation signal and has previously been reported to interact with the nuclear import protein importin-α to regulate meiotic spindle pole organisation, so the localisation of GOLGA2 to the nucleus was not surprising [23]. However, the localisation of JAM-B to the nucleus was unexpected.

**Figure 6:**
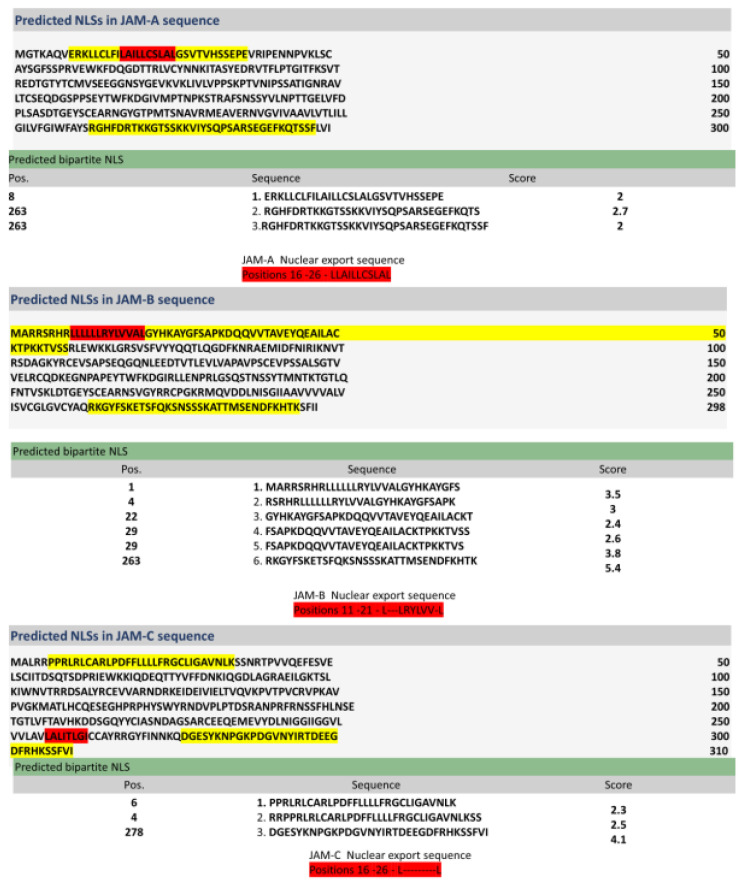
Nuclear localisation and export signals for the junctional adhesion molecules.

Facilitated transport of proteins across the nuclear pore proteins requires the protein to have a nuclear localisation and nuclear export signal. Using the online bioinformatic software cNLS Mapper and the Nuclear Export online software NetNES 1.1 Server, we confirm that JAM-B protein has NLSs at positions Met1-Ser59 and Arg220-Lys294 (Figure 6) and a nuclear export sequence that lies within the Met1-Ser59 nuclear localisation signal at positions Leu11-Leu21 (Figure 6) [21].

### 2.5. Total JAM-B Protein Fluorescence Is Not Affected by Inflammatory Stimuli, but JAM-B Nuclear Localisation Is Enhanced by Inflammatory Stimuli and Inhibition of Poly-Ubiquitination

**Figure 7:**
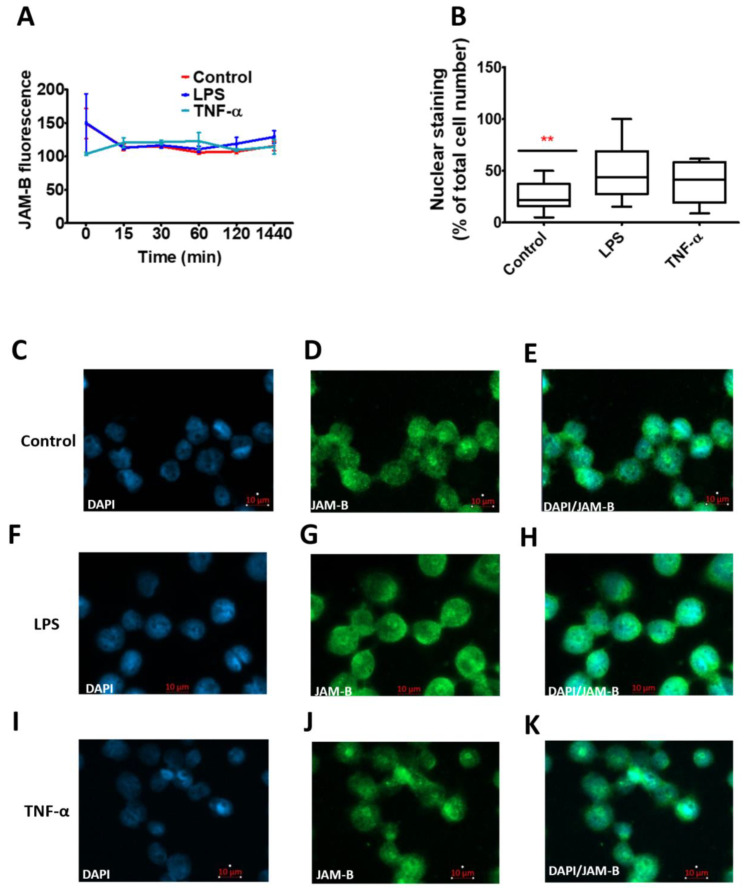
The effects of 10 ng/mL LPS and TNF-α after 24 h incubation on THP-1 macrophage cellular and sub-cellular JAM-B protein expression. (**A**) Time course assessment of total cellular JAM-B protein expression using immunofluorescence; (**B**) percentage of JAM-B expression in the punctate loci of the nucleus; (**C**–**E**) DAPI, JAM-B and composite image in control-treated samples; (**F**–**H**) DAPI, JAM-B and composite image in LPS-treated samples; (**I**–**K**) DAPI, JAM-B and composite image in TNF-α-treated samples. ** indicates *p* ≤ 0.01 in comparison to controls.

The effect of the inflammatory stimuli LPS and TNF-α on JAM-B protein expression was also examined using THP-1-macrophages at various time points using immunofluorescence. No differences in the total JAM-B expression levels were observed over the time course in response to inflammatory stimuli (Figure 7A). However, LPS- and TNF-α-treated cells showed an increase in punctate nuclear expression of JAM-B, which was significantly different from the control in the LPS-treated cells (χ^2^ (2) = 7.6), *p* = 0.02) (Figure 7B). The differences in the staining can also be visually observed in Figure 7C–E for the control, Figure 7F–H for LPS and Figure 7I–K for TNF-α.

**Figure 8:**
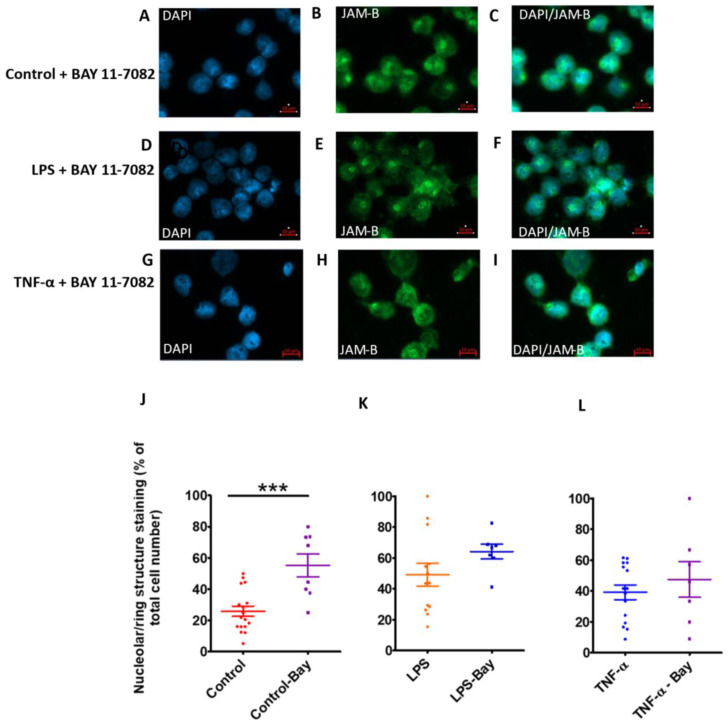
The effects of the NF-κB inhibitor Bay-11-7082 (10 µM) after 24 h treatment on JAM-B subcellular localisation in THP-1-differentiated macrophages. (**A**–**C**) DAPI, JAM-B and composite image in control-treated samples; (**D**–**F**) DAPI, JAM-B and composite image in LPS-treated samples; (**G**–**I**) DAPI, JAM-B and composite image in TNF-α-treated samples. (**J**) JAM-B punctate nuclear localisation quantification showing significant increase in JAM-B expression in the nucleus in the control plus Bay-11-7082-treated samples. (**K**) JAM-B punctate nuclear localisation quantification showing significant increase in JAM-B expression in the nucleus in the LPS plus Bay-11-7082-treated samples. (**L**) JAM-B punctate nuclear localisation quantification showing no significant increase in JAM-B expression in the nucleus in the TNF-α plus Bay-11-7082-treated samples. *** indicates *p* ≤ 0.001.

Protein ubiquitination has been shown to not only regulate protein stability but also its localisation, function and interaction with other proteins [34,35]. To further investigate the mechanisms underpinning JAM-B punctate nuclear localisation, LPS- and TNF-α-treated cells were also treated with Bay-11-7082 [34,35]. Although the inhibitor had no significant effect on JAM-B gene expression in the control unstimulated cells (Figure 8), at protein level using immunofluorescence, there was a significant increase in the punctate nuclear staining of JAM-B protein in the control samples treated with the inhibitor in comparison to controls without the inhibitor (U = 18.0, *p* = 0.003, *n* = 8) (Figure 8A–C,J). Similarly, while at gene expression, the inhibitor reduced gene expression, in the LPS- (Figure 8D–F,K) and TNF-α-treated sample (Figure 8G–I,L), Bay-11-7082 enhanced the punctate nuclear localisation, although this was only significant in the LPS-treated group.

### 2.6. Identification of JAM-B Protein in THP-1 Monocytes Using Immunoblotting

**Figure 9:**
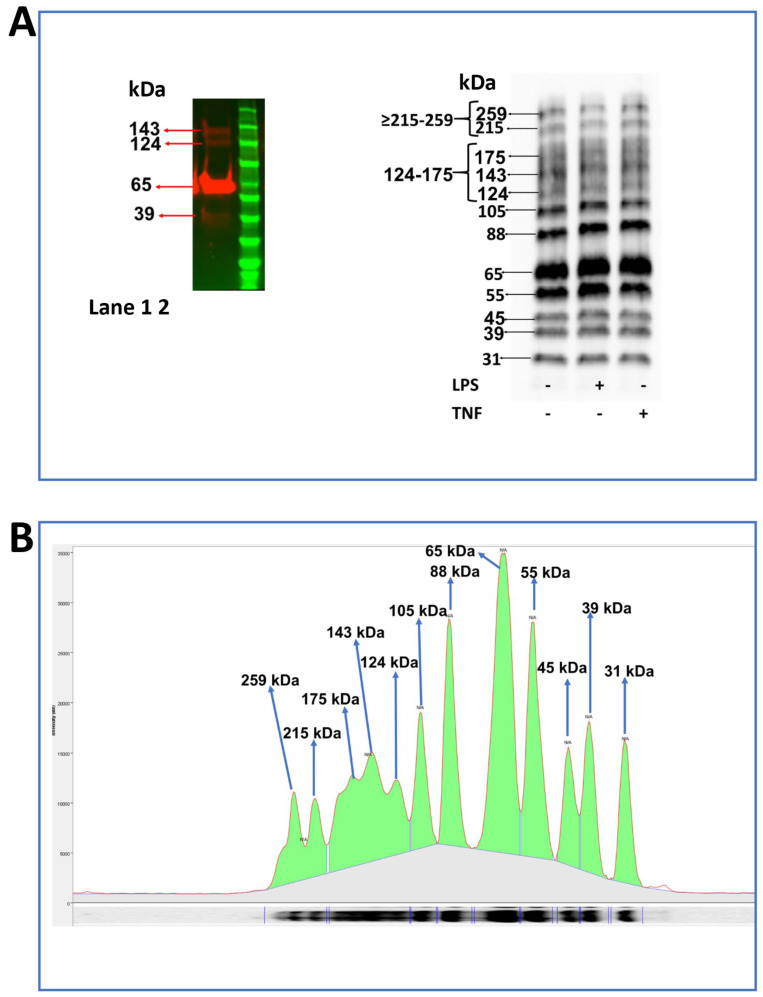
Western blot probing for JAM-B protein in samples of recombinant JAM-B protein and THP-1 monocyte whole cell lysates. All samples were separated by SDS-PAGE under reducing conditions using DTT. (**A**) Recombinant protein (**left panel**, lane 1) run together with protein molecular weight marker (**left panel**, lane 2) and cell lysates from unstimulated (**right panel**) and LPS- (**right panel**) or TNFα- (**right panel**) stimulated THP-1-cultured cells (**right panel**); (**B**) example chromatogram demonstrating band molecular weights and grouping to aid quantification. Representative images of more than three technical replicates.

We next carried out immunoblotting of Dithiothreitol (DTT)-reduced SDS-PAGE gels to examine specific recognition of JAM-B by the mouse monoclonal antibody using the recombinant JAM-B protein (R-JAM-B) (#1074-VJ-050- R & D Systems, Abingdon, UK) and THP-1 whole cell lysates. The recombinant protein (Phe29—Asn236) was devoid of the signal peptide (Met1—Ser29) and the Ile237–Ile 298 peptide, thus lacking 88 amino acids. For the recombinant protein, the blot gave a strongly stained band at 65 kDa, which corresponds with the 64–70 kDa band expected for the recombinant homodimer according to the antibody manufacturer [36]. A very faint lower-molecular-weight band at 39 kDa and two faint higher-molecular-weight bands at 124 and 142 kDa were also visible (Figure 9A). The origin of these bands is not clear, although they may represent dimers and monomers of JAM-B or JAM-B species resulting from post-translational modification, such as protein cleavage. These bands (39, 65, 124 and 142 kDa) were also observed in THP-1 cell lysates along with further bands at 31, 45, 55, 88 and 105 kDa and a group of fainter, more diffuse bands at 175, 215 and 259 kDa (Figure 9B). This pattern of JAM-B staining closely resembles that previously observed in Hela cells where the 45, 55, 64–68, 88 and 105 kDa bands were observed (Supplementary Table S4) [37]. The 31, 55 and 88 kDa bands have also been observed in mouse brain and heart tissue, with the 45 and 55 kDa also being detected in mouse lung tissue (Supplementary Table S4) [37]. In CHO cells over-expressing hJAMB, Cunningham et al. reported a strong band at approximately 48 kDa, which we believe to correspond to the 45 kDa band observed here and by most of the JAM-B antibody manufacturers reported in Supplementary Table S4 [24]. In a report by Cunningham, further fainter bands can be seen at 28, 31, 55, 66 and above 250 kDa, in agreement with our observations and also consistent with the bands observed in a blot image from the JAM-B antibody manufacturer ProSci [24].

All these bands were also detected in cells after treatment with and without inflammatory stimuli (Figure 9A, right panel). High-mass bands as observed at 215 and 259 kDa are common in proteins that have been post-translationally modified by ubiquitination, SUMOylation and acetylation. As the bands between 124 and 175 kDa and those between 215 and 259 kDa were in areas of the blot with diffuse but significant staining between bands, for the purpose of quantification, they were integrated in groups (Figure 9A, right panel).

### 2.7. Distinct Expression Profile of JAM-B Protein Species in Sub-Cellular THP-1 Cell Compartments and JAM-B Post-Translational Ubiquitination

**Figure 10:**
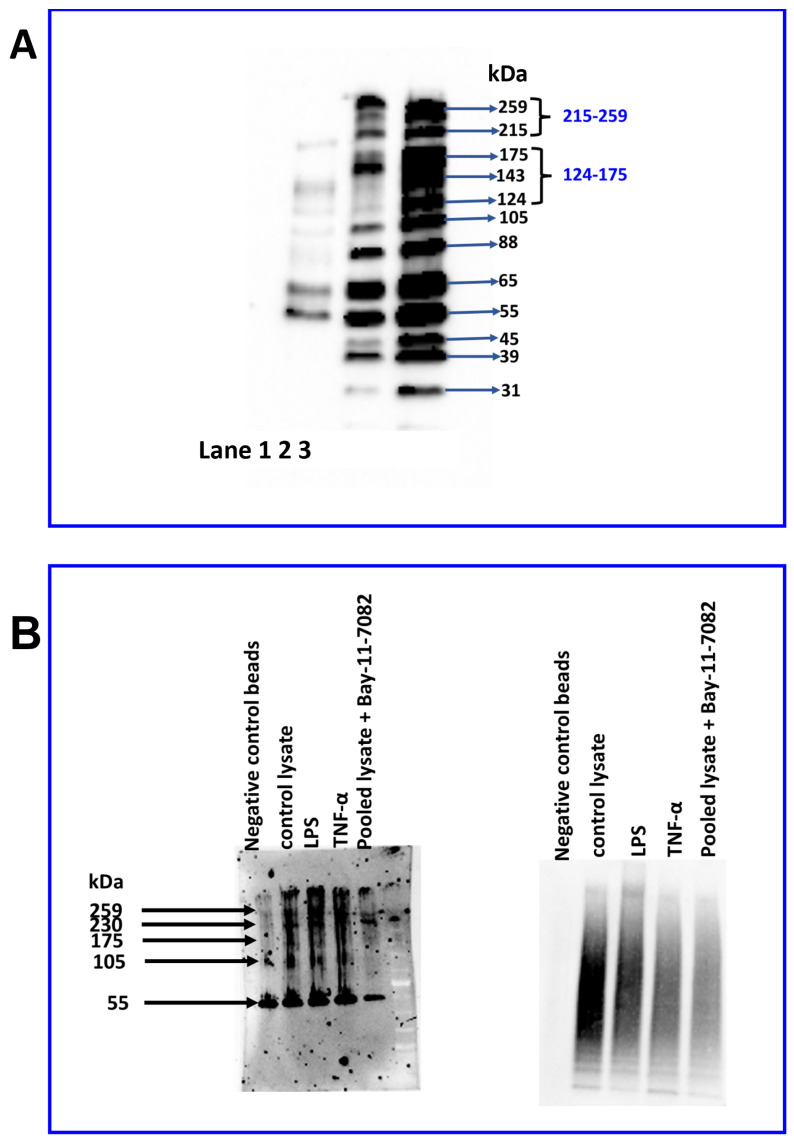
Immunoblots of JAM-B in lysates of THP-1 monocyte whole cells, nuclear and cytosolic sub-fractions of THP-1 cells with and without treatment with 10 µM Bay-11-7082 inhibitor. (**A**) Immunoblot of nuclear (lane 1), cytoplasmic (lane 2) and whole cell lysates (lane 3) probed with anti-JAM-B antibodies. (**B**) Immunoblots of ubiquitin-enriched samples of whole cell lysates prepared by immunoprecipitation probed with anti-JAM-B antibodies (**left panel**) and anti-ubiquitin antibody (**right panel**). Negative control beads denote samples obtained following the immunoprecipitation procedure with negative control beads; control lysate was ubiquitin-captured samples from unstimulated/untreated cells, or cells treated with either 10 ng/mL LPS or 10 ng/mL TNF-α are ubiquitin-captured lysates from cells treated with inflammatory stimuli (LPS or TNFα) and in samples pooled from Bay-11-7082 (10 µM)-treated control and LPS- and TNF-α-treated samples.

As immunostaining indicated sub-cellular localisation of JAM-B, we further characterised the expression profile of JAM-B species in nuclear (Figure 10A, lane 1) and cytosolic (Figure 10A, lane 2) cellular subfractions. While similar to whole cell lysate (Figure 10A, lane C), all JAM-B species were detected in the cytosolic lysates, the 143 and the 124 kDa bands were very faint (Figure 10A). Instead, these bands (143, and 124 kDa) were detected in the nuclear lysate, which also contained the 215 band (Figure 10A). The strongest bands observed in the nuclear lysates were the 55 and 65 kDa bands. In contrast to the cytosolic lysate, in the nuclear lysates, the 259, 105, 88, 45, 39 and 31 kDa bands were either very faint or undetectable, indicating differential expression of the JAM-B species in the cytosol and nucleus.

Subsequently, we performed ubiquitin enrichment immunoprecipitation (IP) assays to determine whether the diffuse nature of the high-molecular-weight protein bands were due to ubiquitination (Figure 10B). In comparison to negative control beads (containing point mutations that prevent ubiquitin binding), whole THP-1 lysate control samples without inflammatory stimuli and those treated with LPS and TNF-α had slightly higher density at ~55 kDa and at 105 kDa and above, which is consistent with JAM-B poly-ubiquitination. Additionally, in the Bay-11-7082-treated pooled sample, the 55 kDa JAM-B band in the ubiquitin IP samples was fainter, although the origin of this band is unclear as the lane containing control beads had also a band at 55 kDa. Together with a faint smear of ubiquitin staining on the right panel in the Bay-11-7082-treated pooled sample, these observations support the notion that Bay-11-7082 inhibits poly-ubiquitination of some ubiquitinylated species but not all ubiquitin linkages [38]. These observations are supported by weaker ubiquitin staining in the inhibitor-treated sample (Figure 10B, right panel). It is worth noting that there is very little staining of the 65 and 88 kDa bands, which suggests that the bands at 65 and 88 kDa JAM-B were not poly-ubiquitinated JAM-B species.

### 2.8. LPS Treatment Reduces the Expression of the 88 and 31 kDa JAM-B Protein Species

**Figure 11:**
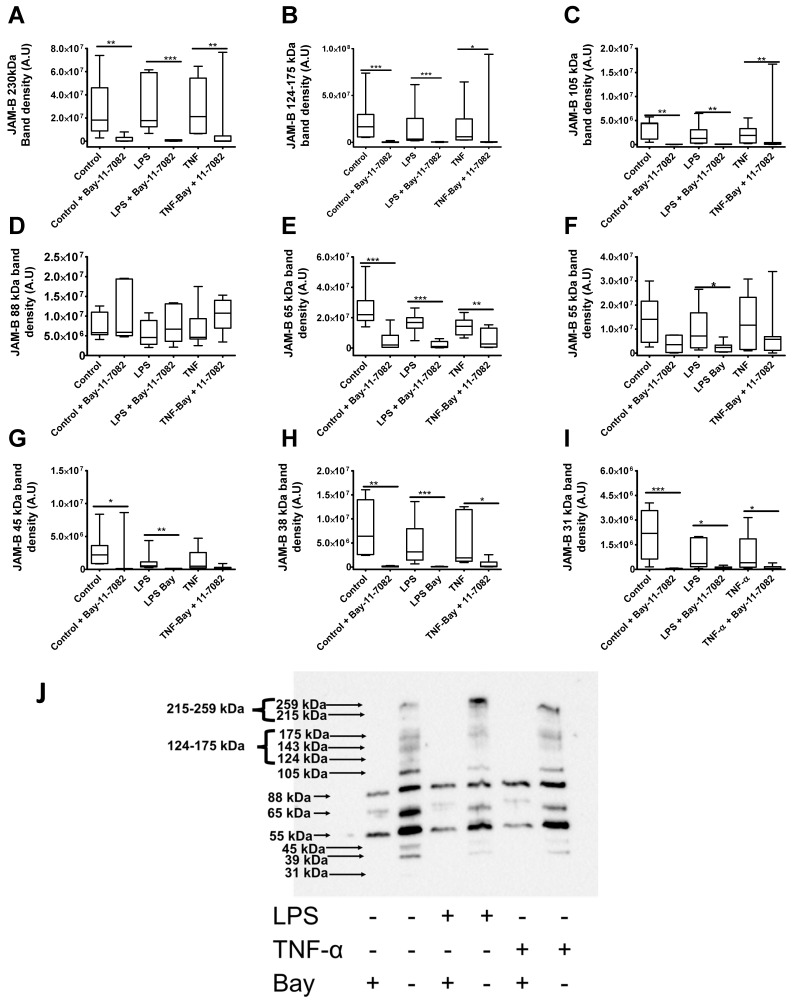
Effects of the Bay-11-7082 inhibitor on the density of JAM-B protein species in THP-1 monocyte whole cell lysates after incubation with and without inflammatory stimuli (relative to total protein on stain-free gel). (**A**) ≥230 kDa bands, (**B**) 124–175 kDa bands, (**C**) 105 kDa band, (**D**) 88 kDa band (**E**) 65 kDa band, (**F**) 55 kDa band (**G**) 45 kDa bands, (**H**) 38 kDa band, (**I**) 31 kDa band and (**J**) blot image showing protein band densities in the Bay-11-7082 inhibitor-treated THP-1 whole cell lysates treated with and without inflammatory stimuli. A.U, arbitrary units; * indicates *p* ≤ 0.05, ** indicates *p* ≤ 0.01, *** indicates *p* ≤ 0.001 after Mann–Whitney U test (*n* = 7). All bands were normalised to the total protein on the stain-free blots.

We next examined the effects of the inflammatory stimuli on the expression of JAM-B species. In line with immunostaining data, there were no statistical differences in the density of the in the majority of the bands between the control and the TNF-α- and LPS-treated groups (*p* > 0.04) (Figure 11). However, the density of the 88 kDa band was slightly lower in the LPS group compared to the control group (U = 0.90, *p* = 0.063, *n* = 17) (Figure 11D), but with only borderline significance, and no differences were observed between the control and the TNF-α-treated groups (U = 137, *p* = 0.80, *n* = 17) (Figure 11D). The density of the of the 31 kDa band was slightly lower in the LPS-treated group in comparison to the control group, and also in the TNF-α-treated group, the density of the 31 kDa band was borderline significantly lower compared to the control, but this was not statistically significant (Figure 11I). There were no statistical differences in the total protein between the control and the LPS group (U = 101, *p* = 0.14, *n* = 17) or between the control and the TNF-α group (U = 120, *p* = 0.41, *n* = 17). Taken together these results suggest that while total JAM-B protein is unaltered by inflammatory stimuli, certain JAM-B species such as the 88 kDa and 31 kDa masses are slightly reduced by LPS.

### 2.9. Bay-11-7082 Increases the Abundance of the 88 kDa JAM-B Species and Reduces the Abundance of Other Higher and Lower Mass JAM-B Species

As the Bay-11-7082 inhibitor has been shown to alter the expression of NF-κB via the proteosome pathway in the context of inflammatory conditions, and here we report that it reduced the densities of JAM-B high-molecular-weight poly-ubiquitinated bands, we next examined the effects of the inhibitor on JAM-B protein species in the presence and absence of inflammatory stimuli. Further incubation of various concentrations of the inhibitor with recombinant JAM-B protein showed that in the protein gel wells containing the inhibitor, the JAM-B bands disappeared at concentrations higher than 2.5 µM Bay-11-7082 inhibitor, indicating that the inhibitor may directly interfere with protein chemistry. This is consistent with reports by Strickson et al., who showed that the inhibitor can form adducts with a variety of proteins [38]. Indeed, in the current study, the Bay-11-7082 inhibitor significantly reduced the expression of the high-molecular-weight JAM-B bands (≥215–259, 142–175 and 105 kDa) (Figure 11A–C) and also the 65 kDa, 55 kDa, 39 kDa and 31 kDa masses, both in the presence and absence of inflammatory stimuli (Figure 11E–I) [29]. Surprisingly, in contrast to this, the JAM-B protein species at 88 kDa was increased by the inhibitor under all conditions, although this was not significant (Figure 11D). The increase in the density of the 88 kDa JAM-B band complements the observations by Strickson et al., who showed increased density of the ubiquitin-conjugating enzyme H7 (UbcH7), also known as ubiquitin-conjugating enzyme E2 L3 (UBE2L3), in response to the Bay-11-7082 inhibitor [38], suggesting that the inhibitor may have physiological effects on this JAM-B protein species. However, the observations that the density of the lower-molecular-weight bands were also reduced or disappeared altogether after treatment with the inhibitor suggests that Bay-11-7082 may also form adducts with lower-molecular-weight JAM-B species or may target other protein processing pathways such as protein cleavage. Indeed, Bay-11-7082 inhibitor has been shown to prevent NLRP3 inflammasome activation by blocking the cleavage of pro-caspase 1 to its active caspase (p20) form by alkylating and inactivating ATPase, which is required for the activation of caspase-1 enzyme [29].

**Figure 12:**
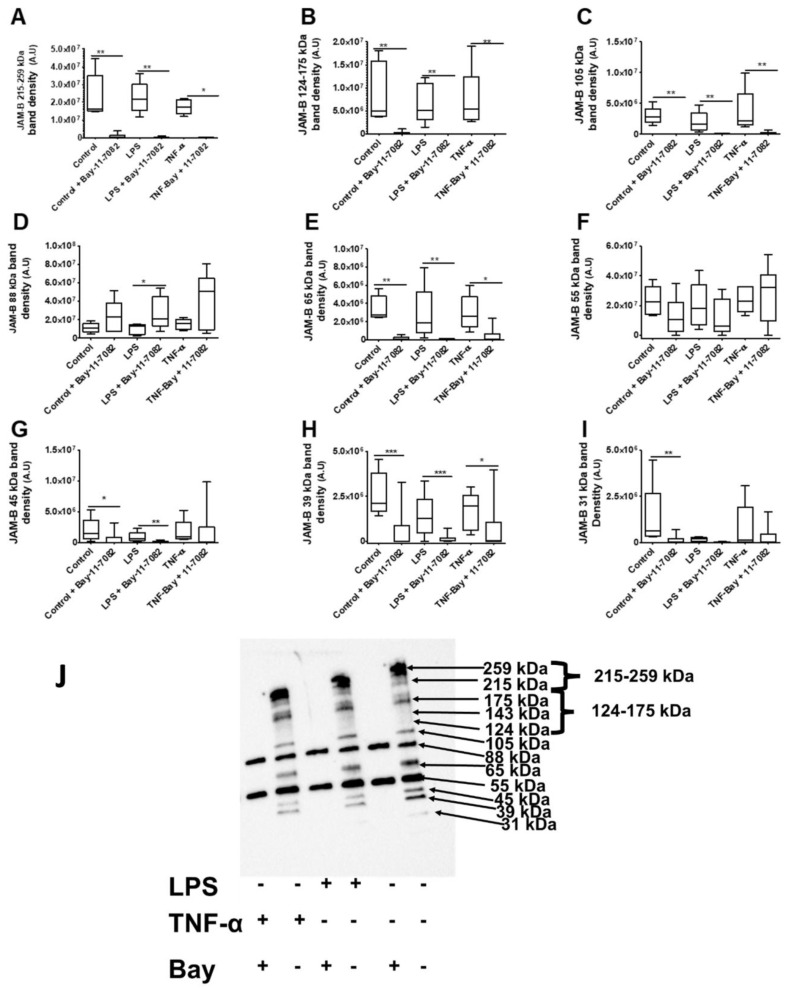
Effects of the Bay-11-7082 inhibitor on the abundance of JAM-B protein species in THP-1 monocyte cytosolic cell lysates after incubation with and without inflammatory stimuli (relative to total protein on stain-free gel): (**A**) 215–259 kDa bands, (**B**) 124–175 kDa bands, (**C**) 105 kDa band, (**D**) 88 kDa band, (**E**) 65 kDa band, (**F**) 55 kDa band, (**G**) 45 kDa band, (**H**) 39 kDa band, (**I**) 31 kDa band and (**J**) blot images showing protein band densities in the Bay-11-7082 inhibitor-treated THP-1 cytosolic cell lysates in the presence and absence of inflammatory stimuli. A.U, arbitrary units; * indicates *p* ≤ 0.05, ** indicates *p* ≤ 0.01, *** indicates *p* ≤ 0.001 after Mann–Whitney U test (*n* = 3).

We next examined the effects of the Bay-11-7084 inhibitor at the sub-cellular level (cytoplasmic and nuclear fractions) in THP-1 monocytes treated with and without inflammatory stimuli. Similar to the observations in the whole cell lysate, in the cytoplasmic lysate, Bay-11-7082 treatment led to significant reductions in the density of the 215–259 kDa, 124–175 kDa, 105 kDa, 65 kDa, 45kDa, 39 kDa and the 31 kDa species in both the presence and absence of inflammatory stimuli (Figure 12A–I). As the total protein staining and loading control were also reduced, statistical comparison between the Bay-11-7082 samples with the untreated groups were carried out tentatively. However, consistent with the whole THP-1 monocyte cell lysate observations, the densities of the 88 kDa bands were increased in the Bay-11-7082 inhibitor-treated groups, although this was prominent in the LPS-treated group (Figure 12D). The densities of the 55 kDa bands were not affected by treatment with the inhibitor in sample treated with and without inflammatory stimuli (Figure 12F). In the nuclear lysates, the inhibitor also reduced the densities of all JAM-B bands and there were no bands whose density was enhanced by the inhibitor (Supplementary Figure S5).

## 3. Materials and Methods

### 3.1. Cell Culture and Treatments

THP-1 monocytic cells were cultured at 37 °C; 5% CO_2_ in RPMI 1640 medium containing 2 mM L-glutamine and supplemented with 10% (*v*/*v*) FBS; 50 IU/mL penicillin and 50 µg/mL streptomycin. Cell densities were maintained between 1 × 10^5^ and 1 × 10^6^ cells/mL. For THP-1 experiments, cells well seeded at 0.5 × 10^6^ cells/mL. Then, 10 ng/mL TNF-α and 1 µg/mL LPS were added as endogenous inflammatory stimuli, and cells were incubated for 24 h. For THP-1-differentiated macrophages, cells were differentiated using 100 ng/mL Phorbol 12-myristate 13-acetate (PMA) for 48 h at similar incubation conditions as the THP-1 monocytic cells. Cells were washed with PBS and then treated with 10 ng/mL TNF-α or 0.1% BSA vehicle control for 24 h. For LPS dose experiments, cells were treated with concentrations ranging from 3.9 µg to 62.5 µg/mL and incubated for 24 h. For the time course experiments, cells were treated with either 10 ng/mL TNF-α or 10 ng/mL LPS with cell harvests at 0, 0.5, 1, 2, 4 and at 24 h. Cells were also treated with the 10 µM Bay-11-7082 inhibitor (Sigma-Aldrich, Gillingham, UK) and incubated overnight. Cells were washed once with PBS and harvested for either RNA extraction or immunostaining. Human primary cells were isolated and verified as described by Day et al. and Layhadi et al. [39,40].

### 3.2. Isolation of Human PBMCs and Primary Leukocytes

Human primary cells were isolated and verified as described by Day et al. (2019) [39]. Blood samples were obtained from healthy volunteers (National Health Service Blood and Transplant; Addenbrooke’s Hospital, Cambridge University Hospital, Cambridge, UK) after ethical approval by the Faculty of Medicine and Health Sciences Research Ethics Committee, University of East Anglia, and the NHS Health Research Authority Ethics Committee. Peripheral blood mononuclear cells (PBMCs) were isolated according to the methodology described by Layhad et al. (2018), where blood was layered over Histopaque-1077 (Sigma Aldrich, Gillingham, UK) and centrifuged at 1000× *g* for 25 min and the top buffy coat was collected [26]. PBMCs were further processed using anti-CD14+ magnetic beads (Miltenyi Biotech, Surrey, UK) to isolate CD14+ monocytes, as described by Day et al. (2019) [27], or were transferred to T75 flasks to adhere for 2 h and cultured at 37 °C, 5% CO_2_ in RPMI 1640 medium containing 2 mM L-glutamine and supplemented with 10% (*v*/*v*) FBS, 50 IU/mL penicillin and 50 µg/mL streptomycin. Granulocyte–macrophage colony-stimulating factor (rHuGM-CSF) (Peprotech, London, UK) was then added and the cells were incubated in the above conditions for 6 days, as described by Layhad et al. (2018) [26].

### 3.3. RNA Extraction

Total RNA was extracted using the RNeasy Mini Kit (Qiagen, Manchester, UK) according to the manufacturer’s instructions. Total RNA concentrations were quantified using a Nanodrop analyser and 1 µg of total RNA was used for cDNA synthesis using the Precision NanoScript Reverse Transcription kit (Primerdesign, Chandler’s Ford, UK) in a final volume of 20 µL according to the manufacturer’s instructions. The cDNA samples were stored at −20 °C and quantitative real-time PCR samples were diluted 1:10 for subsequent quantitative PCR.

### 3.4. Quantitative PCR

Intron-spanning primers were designed using the PubMed primer designing tool (https://www.ncbi.nlm.nih.gov/tools/primer-blast/ (accessed on 26 March 2018)) and checked for specificity using BLAST (https://blast.ncbi.nlm.nih.gov/Blast.cgi (accessed on 26 March 2018)) (Table 1). A test reverse transcription was carried out without the reverse transcriptase enzyme, which gave no product in the after conventional PCR followed by gel electrophoresis PCR, thereby demonstrating that the primers did not amplify genomic DNA.

Primers were synthesised by Integrated DNA Technologies (IDT, Leuven, Belgium). Gene expression analysis was carried out using the VIIA™ 7 PCR System (Life Technologies, Paisley, UK) in a final reaction volume of 10 µL, and composed of 1× ImmoMix PCR MasterMix (Bioline, London, UK), 0.6 × SYBR Green (0.06 µL of 100× stock), ROX reference dye (175 nM), magnesium chloride (0.5 mM), BSA (50 µg/mL) and 10 nM forward and reverse primers. The following PCR cycling conditions were used: initial denaturation at 95 °C for 10 min, followed by amplification and data acquisition at 95 °C for 15 s and annealing/extension at 60 °C for 1 min for 40 cycles and a melt curve to determine primer specificity. All samples were run in triplicate with no template control for each gene. JAM-B PCR product was run on an agarose gel to confirm specificity and the product was excised and sent to for sequencing (Eurofins, Cambridge, UK) to verify that it is specific and aligns to JAM-B transcripts. Sequence alignment was carried out using UGENE software (v38.1) [41]. The geometric mean of CT values for YWHAZ and ACT-β was used for gene expression normalisation after ensuring their stability in the presence of treatments.

### 3.5. Immunostaining

Cells were fixed by incubation in ice-cold methanol for 10 min at −20 °C, after which they were washed twice with PBS and once with TBS for 5 min. Cells were blocked in TBS containing 5% goat serum and 2% BSA for 1 h. Cells were then incubated in primary antibodies overnight at 4 °C for the mouse anti-human JAM-B monoclonal antibody (also used in a study by Jael et al.) (Santa Cruz, Dallas, TX, USA, Cat # sc-293496) or at room temperature for 1 h for the rabbit anti-human JAM-B polyclonal antibody (validated in JAM-B knockout studies by Redmond et al., 2017) (Thermo Fisher Scientific, Hemel Hempstead, UK, Cat # PA5-21576), each at 1:250 dilution, and they were then washed thrice for 5 min each in TBS [42,43]. For JAM-A, an anti-JAM-A mouse monoclonal antibody was used (Santa Cruz, Dallas, TX, USA sc-53623-) at a dilution of 1:250. Cells were treated with goat anti-mouse secondary antibody (A32723-Life Technologies/Invitrogen, UK) or goat anti-rabbit secondary antibody (Life Technologies/Invitrogen, Paisley, UK, Cat # A-11012-), each at a dilution of 1:2000 (a no secondary antibody was used as a control for non-specific staining), and incubated at room temperature for 1 h. They were then washed thrice for 5 min each before mounting with Fluoromount™ Aqueous Mounting Medium (Sigma, Gillingham, UK) and they were analysed using the Zeiss Widefield Fluorescence microscope interphase with the Zen software (2.3 Lite (blue edition), Zeiss, Oberkochen, Germany). Punctate nuclear localisation was determined by manual counting of JAM-B staining within the DAPI stained cells and calculated as a percentage of total DAPI stained cells.

### 3.6. Protein Extraction and Western Blotting

Proteins were extracted from whole cell lysates using a mixture of 1 × RIPA buffer with protease inhibitor cocktail (Merck Life Science, London, UK), and cytosolic and nuclear cell components were extracted using the NE-PER™ Nuclear and Cytoplasmic Extraction Reagents according to the manufacturer’s instructions (Life Technologies/Invitrogen, UK) also with protease inhibitor cocktail added prior to the addition of the Cytoplasmic Extraction Reagent I (CER I). Protein quantification was carried out using the Pierce™ BCA Protein Assay Kit according to the manufacturer’s instructions (Life Technologies/Invitrogen, UK). Lysates were kept at −80 °C until immunoblotting. Then, 20 µg of whole cell lysates or 15 µg of cytosolic and nuclear lysate were mixed with Laemmli buffer (Bio-Rad, Kidlington, UK), premixed with β-mercaptoethanol (Bio-Rad, UK) or Dithiothreitol (DTT) and boiled at 95 °C for 10 min. Lysates were electrophoresed on 4–15% Mini-PROTEAN^®^ TGX Stain-Free™ Protein Gels (Bio-Rad, UK) and proteins were transferred to 0.45 µm PVDF Transfer Membrane (Thermo Fisher, USA). Gels were activated prior to transfer and membranes were also activated after transfer to obtain total protein transferred. Membranes were washed twice for 5 min with Tris-Buffered Saline, 0.1% Tween^®^ 20 Detergent (TBST) and blocked with 5% milk powder (Marvel Skimmed Milk, UK). Membranes were washed thrice for 5 min each with TBS-T and incubated in primary antibodies overnight at 4 °C for the mouse anti-JAM-B monoclonal antibody (Santa Cruz, USA, Cat # sc-293496) or room temperature for 1 h for the rabbit anti-JAM-B polyclonal antibody (Thermo Fisher Scientific, UK, Cat # PA5-21576), each at 1:200 dilution. Membranes were then washed thrice for 5 min each and incubated in either goat anti-mouse IgG (H + L) Cross-Adsorbed Secondary Antibody, HRP conjugate (Life Technologies/Invitrogen, UK, Cat # G-21040) or goat anti-rabbit IgG antibody, HRP-conjugate antibodies (Merck Life Science UK Ltd., Cat # 12-348) at 1:2000 dilution. Membranes were washed thrice for 5 min each and developed using Clarity and Clarity Max ECL Western Blotting Substrates (Bio-Rad, UK). Membrane images were obtained and analysed using ChemiDoc™ MP System with Image Lab™ Software (6.1.0.07, Bio-Rad, UK). Protein quantity was quantified relative to total protein on stain-free blot lanes. It is noteworthy that although the same amount of protein was loaded for all treatments, in the Bay-11-7082-treated groups, total protein in the stain-free gels was reduced. This effect was only observed in cell culture conditions but not when the inhibitor was added directly to the protein lysate. The JAM-B mouse monoclonal antibody (sc-293496) was further validated using recombinant mouse JAM-B/VE-JAM Fc chimera protein (R & D Systems, UK, Cat # 88-VJ-050).

### 3.7. Ubiquitin Enrichment and Immunoprecipitation

To verify JAM-B ubiquitination, cells were ubiquitin-enriched using the signal-seeker ubiquitin enrichment kit according to manufacturer’s instructions (Universal Biologicals/Cytoskeleton. Inc., Cambridge, UK). Briefly, cells were plated at 1 × 10^6^/mL in four six-well plates, as described above. Cells were treated with vehicle control buffer, TNF-α (10 ng/mL) and LPS (10 ng/mL). The fourth plate contained duplicates of control and inflammatory stimuli, which were pooled for treatment with negative control beads. Additionally, six further wells were treated with either control, LPS or TNF-α as well as the 10 µM Bay-11-7082 inhibitor in duplicates. After overnight incubation, proteins were isolated and enriched for ubiquitin according to the manufacturer’s instructions. The ubiquitin-enriched samples and negative control bead samples were subjected to immunoblotting for JAM-B, as described above. The blots were further immunostained using a mouse anti-ubiquitin-HRP conjugated antibody (Universal Biologicals/Cytoskeleton. Inc., UK, Cat# CUB02) overnight at 4 °C.

### 3.8. Nuclear Export and Localisation and Motif Prediction

For most proteins, the import and export of proteins from cellular compartments to the nucleus requires the protein to have a nuclear localisation and nuclear export sequence. Nuclear localisation sequence prediction was carried out using the importin α-dependent nuclear localisation signal software (cNLS Mapper—http://nls-mapper.iab.keio.ac.jp/cgi-bin/NLS_Mapper_help.cgi (accessed on 3 May 2021) [44]. Nuclear export sequence prediction was carried out by the Nuclear Export bioinformatic software (NetNES 1.1 Server) [45]. External cues such as inflammatory stimuli can determine whether a certain protein is localised to the nucleus and how that protein is involved in down-stream signalling pathways. The computational biology online resource for determining short linear motifs (SLiMs) was used to characterise candidate motifs that may provide insights into JAM regulation and functionality (http://elm.eu.org (accessed on 14 June 2021)) [46].

### 3.9. Statistics

All data were prepared and processed using Microsoft Excel, and statical analyses were carried out and graphs drawn using GraphPad Prism version 5.0 for Windows (GraphPad Software, San Diego, CA, USA, www.graphpad.com (accessed on 10 June 2022)). Comparison between three groups or more were carried out using Kruskal–Wallis One-way analysis of variance with Dunn’s multiple comparison test with the data presented as chi squared values (χ^2^ (2)) and *p* values. For comparison between two groups, the Mann–Whitney Test was used, with data presented as medians (mdn) and quartile ranges, and the significance estimates were presented as Mann–Whitney U (U) alpha values along with *p* values. In the graphs, * represents *p* ≤ 0.05, ** represents *p* ≤ 0.01, *** represents *p* ≤ 0.001 and **** represents *p* ≤ 0.0001. All experiments were repeated at least three times unless otherwise stated.

## 4. Discussion

We aimed to characterise the expression of JAM-B in leukocytes and determine mechanisms through which it is regulated under host- and pathogen-derived inflammatory stimuli. Contrary to the literature, we demonstrate that JAM-B is expressed in leukocytes and that the expression of JAM-B gene is regulated via NF-κB-dependent pathways in response to both host- and pathogen-derived inflammatory stimuli. Despite increased mRNA levels in response to both LPS and TNF-α, total protein quantity remained unaltered by these inflammatory stimuli. However, the densities of the 88 kDa, 55 kDa and 31 kDa JAM-B species were slightly decreased by LPS treatment. Additionally, JAM-B punctate nuclear localisation was increased after LPS treatment and further increased by an inhibitor of poly-ubiquitination. These results indicate that JAM-B is regulated at the post-translational level via ubiquitin-dependant mechanisms, and it is possible that this affects its stability, subcellular localisation and response to inflammatory stimuli.

Junctional adhesion molecules are a type of tight junction proteins which have been shown to play a role in the maintenance of cell–cell interactions. Three main isoforms of JAMs exist, namely, JAM-A, JAM-B and JAM-C. The former has been shown to play a role in leukocyte adhesion to the endothelium during inflammation and as receptor for viruses, and as such has been implicated in communicable and non-communicable diseases [4,47]. Mechanistic studies have suggested that a JAM-A protein on leukocytes can interact with another JAM-A protein on the endothelium, while JAM-C localised on leukocytes can interact with JAM-B on the vascular endothelium or an integrin dimer, integrin α4β1 (very late antigen-4/VLA-4) [24]. Interestingly, the current literature suggests that JAM-B is mainly expressed in the endothelium rather than in leukocytes. Here, we show that while both JAM-A and JAM-B are expressed at the mRNA and protein level in THP-1 monocytes and THP-1-differentiated macrophages, JAM-C is only expressed in the former and all JAMs are expressed at the mRNA level in human primary monocytes and macrophages as well as PBMCs. We further show that while the level of transcription JAM-A is negatively regulated by TNF-α stimulation, JAM-B is positively regulated by TNF-α, suggesting that these JAMs may be differentially regulated under inflammatory conditions. The observation that the expressions of JAM-A and JAM-C are not altered in inflamed cells is in agreement with the report by Ueki et al. (2008), who provided evidence that in human aortic endothelial cells and neonatal dermal lymphatic endothelial cells, these two JAMs are not altered by inflammatory stimuli at either the protein or gene expression level, and suggested that their contribution to leukocyte adhesion is independent of inflammatory stimuli [48]. In contrast, here we show that in THP-1 monocytes, JAM-B gene expression is strongly up-regulated by inflammatory stimuli. The NF-κB inhibitor Bay-11-7082 prevented the LPS- and TNF-α-induced increases in JAM-B gene expression, indicating that JAM-B transcription is regulated via the NF-κB pathway. Similar to the transcriptional regulation of NLRP3 by NF-κB, it is possible that while at the gene expression level, NF-κB may regulate JAM-B, and at the protein level, other mechanisms may be involved [29,49]. Other pathways that may be affected include the caspase cleavage pathways and NLRP3 inflammasome signalling [28,30]. The inhibitor has indeed been shown to directly inhibit the NLRP3 inflammasome at the protein level, and as such, direct inhibition of JAM-B protein expression by the inhibitor is also possible [29].

As JAM-B has been shown to localise to cellular junctions, the observations reported here of JAM-B distribution to the nucleus, as demonstrated by both immunostaining and immunoblotting, are novel and surprising. Interestingly, although not mentioned in the study by Ueki et al., JAM-C protein seemed to also be expressed in punctate cellular locations in aortic endothelial cells and seemed to be increased after TNF-α stimulation [48]. In the current study, the punctate nuclear staining of JAM-B was similar to that observed for the NLRP3 inflammasome protein—apoptosis-associated speck-like protein containing a caspase recruitment domain (ASC) in PBMCs and THP-1-derived cell lines, which were also enhanced by inflammatory stimuli [50].

In the current study, we also show that although total protein expression was not altered, punctate nuclear JAM-B expression was increased in cells treated with LPS and TNF-α, suggesting that JAM-B protein localisation is modulated by inflammatory conditions. The discord in JAM-B mRNA and protein expression may indicate substantial control of JAM-B protein levels at post-translational levels to limit its accumulation, as observed with an inflammasome protein, NLRP3 [39,40,51]. Indeed, we show that in control and LPS- and TNF-α-stimulated cells, JAM-B was poly-ubiquitinated.

Bay-11-7082 has been shown to inhibit proteasomal degradation of hypoxia-inducible factor 1α (HIF1α) by inhibiting ubiquitin conjugation enzymes E2 L3 (UBE2L3), UBCH7 and UBC13, as well as the E3 ligase linear ubiquitin assembly complex (LUBAC), so that formation of the Lys^63^-linked (k63) poly-ubiquitin chain, which aids protein degradation, is hindered. The inhibition of the NF-κB pathway by Bay-11-7082 has also been shown to be modulated via alterations of ubiquitin conjugation enzymes [38]. It is possible that Bay-11-7082 may directly or indirectly regulate JAM-B expression in mechanisms involving the ubiquitin conjugation enzymes. We hypothesised that if JAM-B was poly-ubiquitinated, Bay-11-7082 should eliminate or reduce the abundance of the higher-molecular-weight bands and increase the density of some lower-mass JAM-B protein species, especially the masses in the region where poly-ubiquitination was not observed. Indeed, the inhibitor reduced the density of the high-mass bands and increased that of the 88 kDa band. Interestingly, the other lower-molecular-weight bands were also substantially reduced. The reasons for this are not clear, but as observed by others, the inhibitor can also prevent the cleavage of proteins such as pro-caspase 1 and gasdermin D (GSDMD) by covalently binding to cysteine residues and inactivating ATPase, which is required in the cleavage of these NLRP3 inflammasome proteins, and prevent the formation of their active counterparts [29].

If JAM-B is subject to regulation by ubiquitination, it should have motifs that aid its recognition by the proteosome for both ubiquitination and de-ubiquitination. This was further verified using the computational biology online resource for SLiMs, which provided possible candidates for the ubiquitination and de-ubiquitination of JAM-B—the Anaphase-Promoting Ubiquitin Ligase Complex APC/C binding Destruction motif (.R..L..[LIVM].), which has been shown to selectively target cell cycle-regulatory proteins for ubiquitin-mediated proteasome degradation, and the USP7/HAUSP MATH domain binding motif ([PA][^P][^FYWIL]S[^P], which aids its interaction with the DUB ubiquitin-specific protease 7 (USP7) for the removal of ubiquitin chains were identified. These motifs have been shown to be critical for pathogen survival and replication in the host as well as in cancer cell development [52]. The pattern of JAM-B staining, especially in the Bay-11-7082-treated samples, suggests expression during the cell cycle (Supplementary Figure S4) and would be consistent with JAM-B regulation by the cell cycle-specific APC/C binding Destruction motif. A further protein blast search also indicated that JAM-B protein has two S/TLQ motifs (BC boxes) which are common in cytokine regulating proteins such as suppressor of cytokine signalling (SOCS), although we could not find the accompanying Cullin RING ligase domains. Nevertheless, mere disruption of the S/TLQ motif has been shown to prevent the interaction of the HIV-1 viral infectivity factor (Vif) with the SOCS–ElonginBC complex, which is otherwise used by the virus to degrade antiviral proteins, Apolipoprotein B Editing Complex (APOBEC3). Thus, it remains to be investigated how these JAM-B motifs are regulated during bacterial and viral infections. While the inhibitor has been shown to reduce K63 poly-ubiquitination chain formation and increase K48 linkages, a recent study has shown that the interaction between K48 and K63 poly-ubiquitination plays a role in both substrate degradation and signal transduction and in particular under inflammatory conditions [39,53]. As Bay-11-7082 has been shown to prevent NF-Κβ nuclear translocation, it was surprising that in control cells, it enhanced the punctate nuclear JAM-B staining in both LPS- and TNF-α-treated cells. It may be possible that the punctate localisation is a result of JAM-B-Bay-11-7082 adducts.

In conclusion, these data suggest that JAM-B is expressed at the mRNA level in leukocytes, including primary human monocytes and macrophages, and in THP-1 monocytes and THP-1 PMA differentiated macrophages at the protein expression level. We further show that as well as localisation to the cytoplasm and the Golgi apparatus, at least in THP-1 cell lines, JAM-B protein is also localised to the nucleus. These findings are contrary to previous studies that have suggested that JAM-B is not expressed in leukocytes, and it points to the possibility of homotypic interaction of JAM-B on leukocytes with JAM-B on the endothelium. The finding that JAM-B is localised to the nucleus is also surprising and contrary to the known role of JAM-B as an adhesion molecule. We further show that JAM-B gene expression is regulated by NF-κB-dependent pathways, and that JAM-B protein function and regulation are not only confined to its primary structure as an immunoglobulin and an adhesion molecule, but may also be subject to post-translational modifications conferred by its interaction with other proteins through its short linear motifs. As the purpose of the work was to characterise the expression and regulation of JAM-B, we have not carried out functional/sequence mutation studies to elucidate specific functions of JAM-B SLiMs. Nevertheless, it is critical to note that SLiMs are the most abundant post-translational modifications that can substantially alter protein function depending on the stress condition and are used by a range of pathogens to increase infectivity and replication within the host, making them an attractive therapeutic target for the treatment of both communicable and non-communicable diseases. Future work should aim to determine the role of these SLiMs in the regulation of the JAMs in health and disease and elucidate why JAM-B nuclear localisation is particularly enhanced under pathogen-derived inflammatory stimuli. Additionally, it would be important to understand the role of JAM-B in inflammatory conditions, for example, by using PBMCs from septic patients.

## Figures and Tables

**Table 1:** Primers sequences and expected product sizes (base pairs).

Gene	Forward Primer	Reverse Primer	Product Size (bp)
JAM-A	5′GGGTGACCTTCTTGCCAACT′3	5′GATGGAGGCACAAGCACGAT′3	142
JAM-B	5′AGGCCTATGGGTTTTCTGCC′3	5′CAAAGGAGACACTCCGACCC′3	144
JAM-B	5′AATTCAGGGAGACTTGGCGG′3	5′TTTCGAGCAACGACCTCACA′3	114
YWHAZ	5′GCAATTACTGAGAGACAACTTGACA′3	5′TGGAAGGCCGGTTAATTTT′3	96
ACT-β	5′GCACCCAGCACAATGAAGA′3	5′CGATCCACACGGAGTACTTG′3	64

## Data Availability

The data that support the findings of this study are contained within the article and the Supplementary Material, and also are available from the corresponding author upon reasonable request.

## Supplementary Materials

The following supporting information can be downloaded at: https://www.mdpi.com/article/10.3390/ijms23158646/s1.
