# Supplementary material for: Transcriptional and Post-Translational Regulation of Junctional Adhesion Molecule-B (JAM-B) in Leukocytes under Inflammatory Stimuli
Source: Int J Mol Sci. 2022 Aug 3;23(15):8646. doi: 10.3390/ijms23158646 (PMC9369439; doi:10.3390/ijms23158646)
Supplement: Supplementary file 1 [file ijms-23-08646-s001.zip › ijms-1792208-supplementary.pdf]

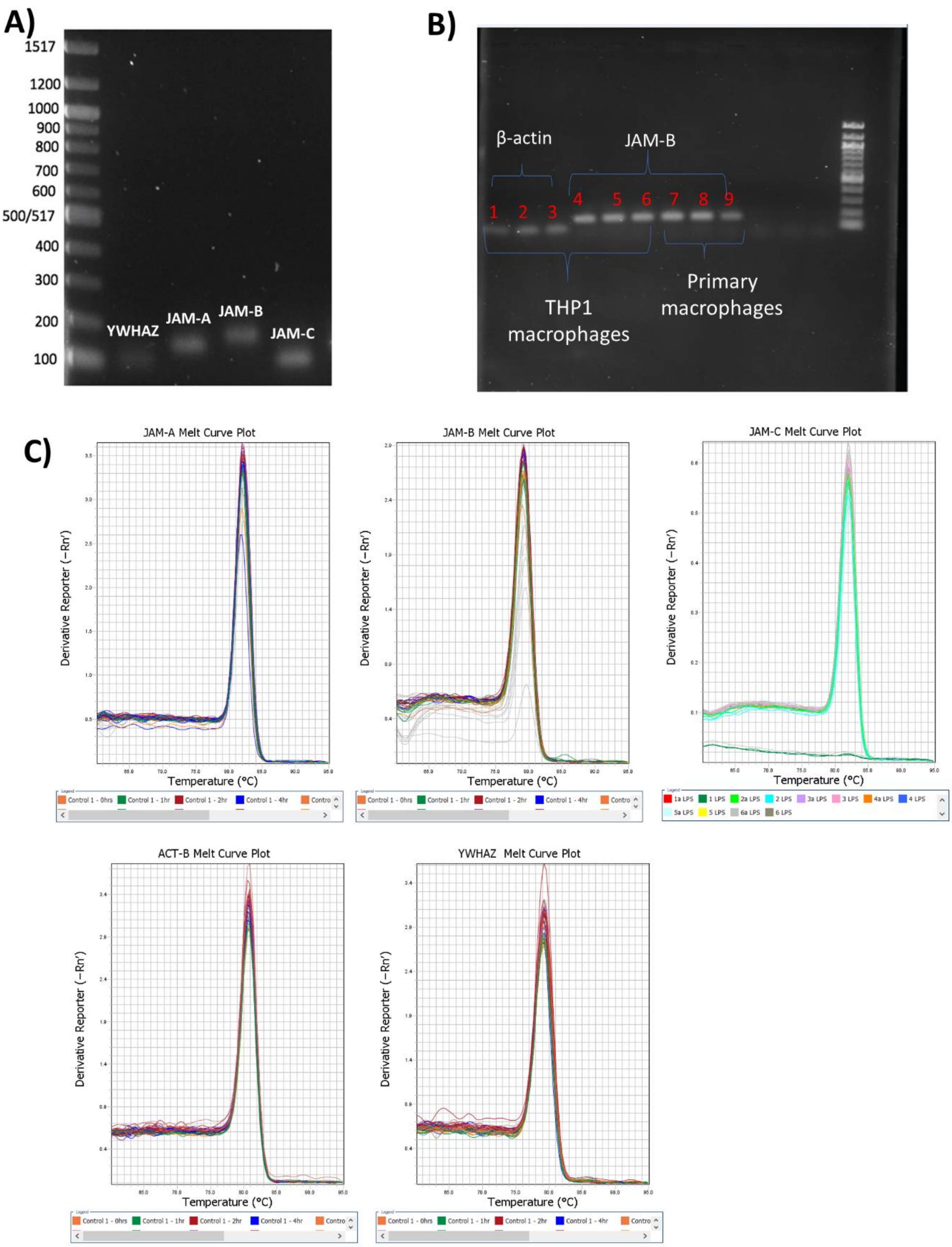

Figure S1. Evidence of primer specificity from gel electrophoresis and PCR melt curves. A) and B) Agarose gels showing single products for JAM-A at 142bp, JAM-B at 144 bp, JAM-C at 114bp, YWHAZ at 96 bp and ACT-β at 64bp. C) melt curves showing single.

curves that provide evidence of primer specificity. Numbers on left side of the gel are molecular markers representing a scale of 100 bp to 2,000 bp. Numbers in figure B are technical replicates with 1-3 representing beta-actin in THP-1 monocytes, 4-6 representing JAM-B in THP-1 monocytes and 7- 9 representing JAM-B in primary macrophages.

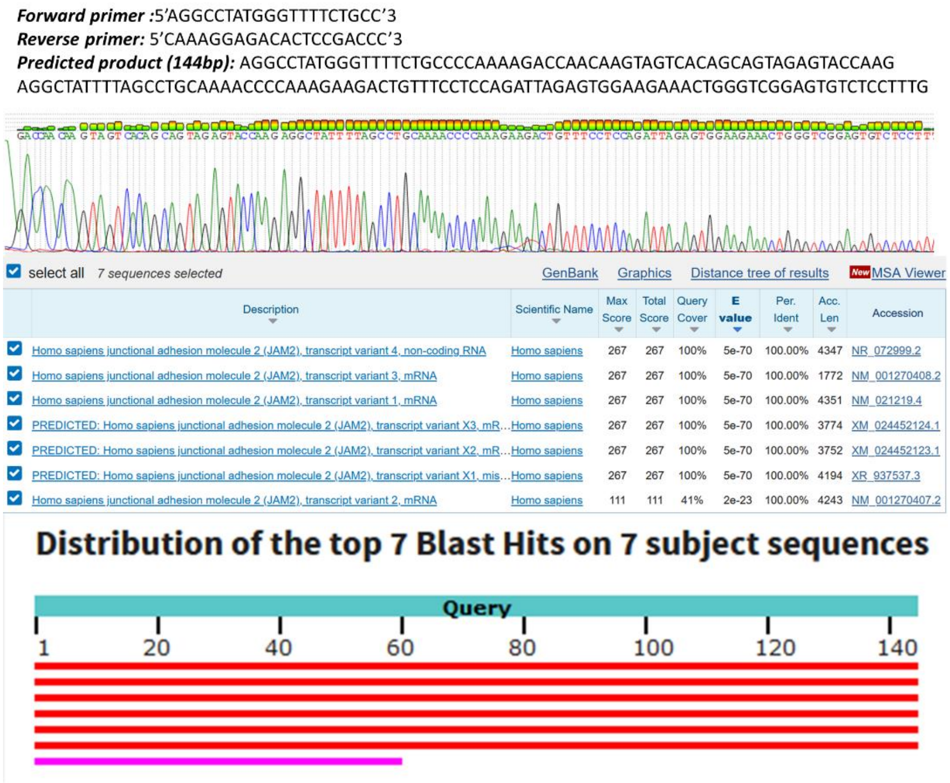

Figure S2. Additional evidence of JAM-B primer specificity. C) Sequencing results for the product (144 bp) from a JAM-B RT-PCR reaction showing a 100% match with the target JAM-B sequence and alignment only with JAM-B transcripts. The 144 bp hJAMB product DNA sequence did not have 100% sequence identity with any non-JAMB DNA or mRNA sequence.

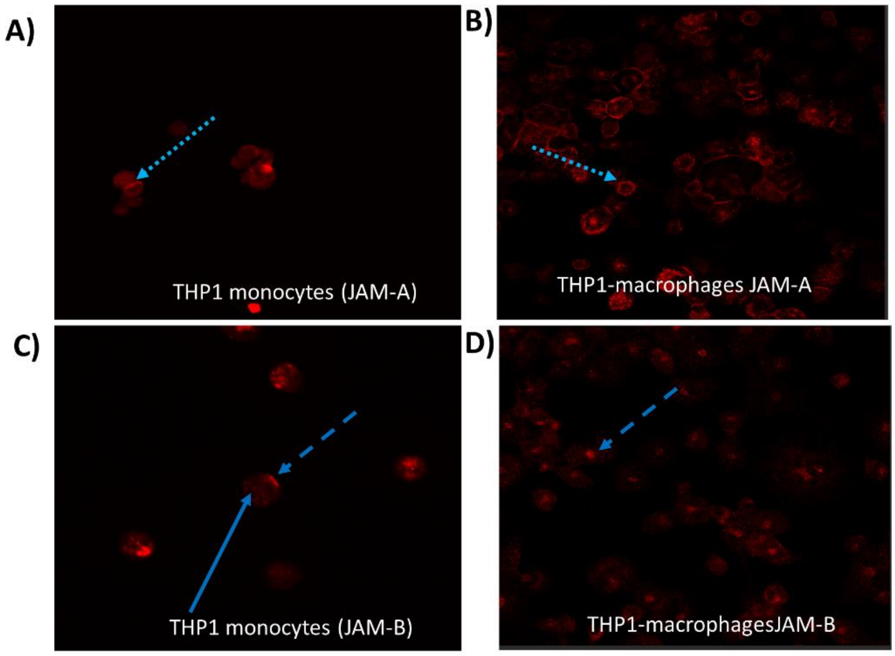

Figure S3. Immunofluorescent staining of JAM-A and JAM-B in intact human THP-1 monocytes and macrophages using a mouse monoclonal JAM-A (sc-53623) and a rabbit polyclonal JAM-B antibody (PA5-21576). A and B) JAM-A protein expression, C and D), JAM-B protein expression, arrows with dotted lines indicate cell junction and cell surface staining, dashed lines indicate polarised staining and solid lines indicate granular staining. Further technical replicates of at least three experiments were carried out as explained in the next sections.

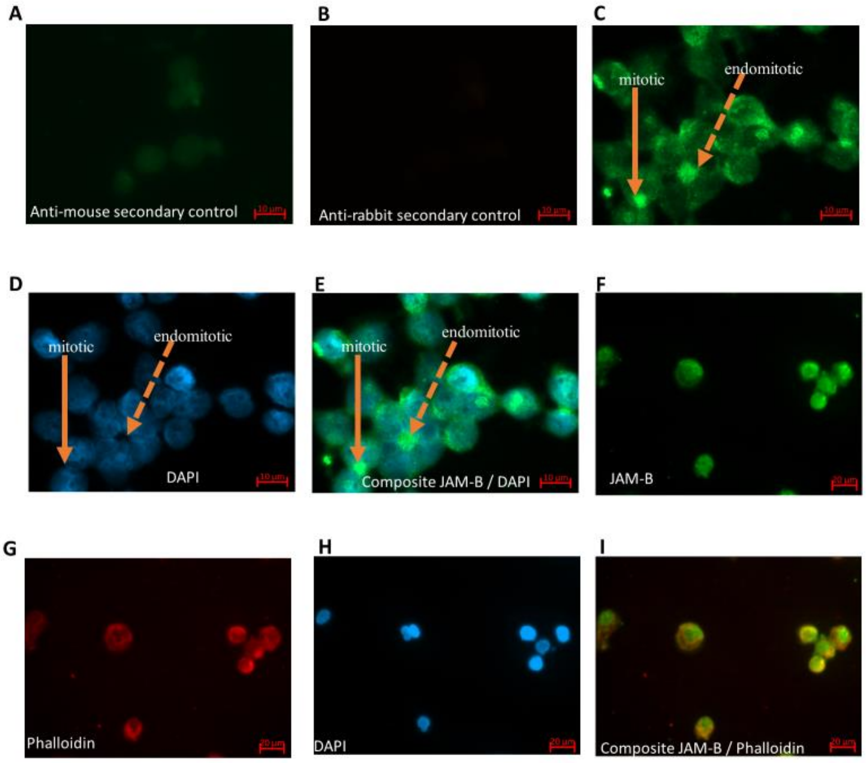

Figure S4. Immunostaining of negative controls for secondary antibodies, JAM-B, DAPI and phalloidin. A) Anti-mouse secondary antibody staining, B) Anti-rabbit secondary antibody staining, C) JAM-B, D) DAPI, E) Composite JAM-B and DAPI, F) JAM-B, G) Phalloidin, H) DAPI and I) Composite DAPI and phalloidin staining.

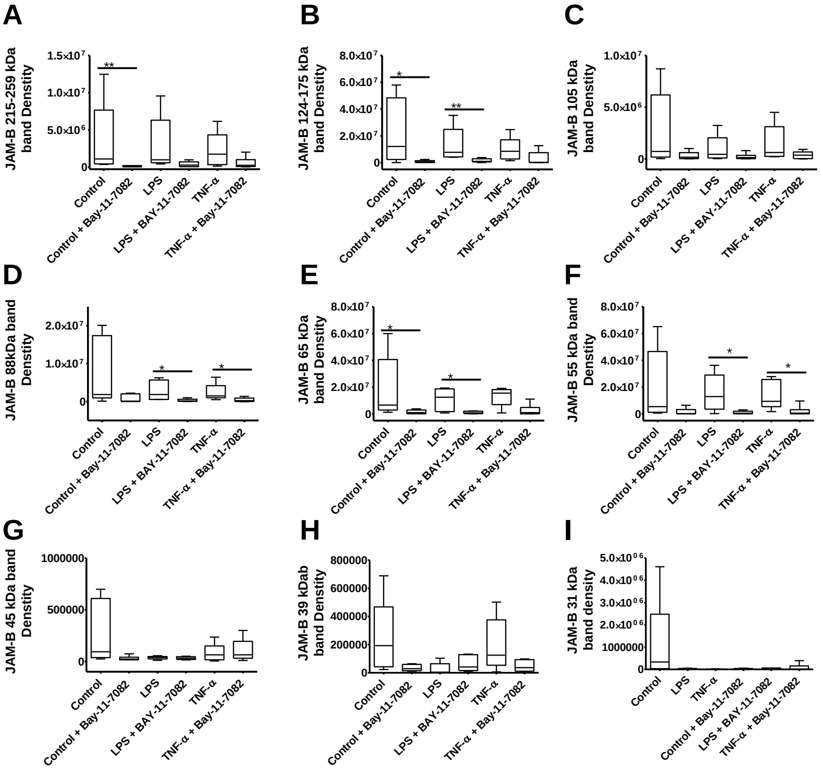

Figure S5. Effects of the Bay-11-7082 inhibitor on the expression of JAM-B protein species in THP-1 monocyte nuclear cell lysates after 24 h incubation with and without inflammatory stimuli (relative to total protein on stain free gel). A) ≥230 kDa band, B) 124-175 kDa band, C) 105 kDa band, D) 88 kDa band, E) 65 kDa band, F) 55 kDa band G) 45 kDa band, H) 39 kDa band and I) 31 kDa band.

Table S1. Relative expression of JAMs in leukocytes against ACT-β and YWHAZ. Data are Mean ± SD.

	JAM-A	JAM-B	JAM-B
**TPH-1 monocytes**	0.93 ± 0.22	0.17 ± 0.09	1.17 ± 0.33
**TPH-1 differentiated macrophages**	0.91 ± 0.14	0.86 ± 0.19	
**Primary monocytes**	11.44 ± 0.31	2.15 ± 0.40	0.38 ± 0.06
**Primary Granulocyte-macrophage colony-stimulate factor macrophages**	5.011 ± 7.35	1.00 ± 0.74	1.01 ± 0.05
**Peripheral blood mononuclear cells (PBMCs)**	6.19 ± 0.37	1.28 ± 0.49	0.21 ± 0.080

Table S2. Fold-changes in JAM gene expression in response to TNF-α and LPS treatments for 24 hours relative to the geometric mean of YWHAZ and Act-β expression. Data are Mean ± SD.

		Control	TNF-α	P value (control vs TNF-α)	LPS	P value (control vs LPS)
**Monocytes**	JAM-A	1.02 ± 0.16	1.07 ± 0.27	0.3865	2.183± 0.99	0.0003
	JAM-B	1.14 ± 0.36	17.14 ± 0.19	0.0001	9.71 ±4.83	0.0001
	JAM-C	1.02 ± 0.15	0.89 ± 0.24	0.1359	0.77 ± 0.43	0.0311
**THP-differentiated macrophages**	JAM-A	1.00 ± 0.08	0.96 ± 0.08	0.2963	NA	NA
	JAM-B	1.10 ± 0.09	1.615 ± 0.28	0.0022	NA	NA

Table S3. Gene expression responses of the JAMs at various time points and to varying LPS doses relative to the geometric mean of YWHAZ and Act-β expression.

	Time (hr)					
	**0 (h)**	**0.5 (h)**	**1 (h)**	**2 (h)**	**4 (h)**	**24 (h)**
**JAM-A**	1.20 ± 0.36	0.83 ± 0.23	0.90 ± 0.30	0.70 ± 0.34	0.68 ± 0.27	0.68 ± 0.26
**P value**		≥0.05	≥0.05	≤0.05	≤0.05	≤0.05
**JAM-B**	0.82 ± 0.41	1.26 ± 0.76	0.85 ± 0.18	2.12 ± 0.61	5.46 ± 2.92	6.37 ± 8.69
**P value**		≥0.05	≥0.05	≥0.05	≤0.001	≤0.05
**JAM-C**	1.06 ± 0.037	0.69 ± 0.17	0.98 ± 0.25	0.82 ± 0.26	1.24 ± 0.31	1.01 ± 0.56
**P value**		≥0.05	≥0.05	≥0.05	≥0.05	≥0.05
	**Dose (ng/ml)**					
	**0**	**3.9**	**7.8**	**31.25**	**62.5**	
**JAM-A**	1.01 ± 0.10	1.27± 0.72	1.45 ± 0.98	1.66 ± 0.95	1.35 ± 1.60	
**P value**		≥0.05	≥0.05	≥0.05	≥0.05	
**JAM-B**	1.08 ± 0.21	5.45 ± 0.98	8.60 ± 4.42	10.41 ± 3.95	10.75 ± 5.04	
**P value**		≥0.05	≤0.05	≤0.0001	≤0.001	
**JAM-C**	1.358 ± 0.90	1.21 ± 0.33	2.80 ± 2.92	1.45 ± 0.33	1.33 ± 0.54	
**P value**		≥0.05	≥0.05	≥0.05	≥0.05	

Table S4. JAM-B molecular species/bands reported for various cell lines and in human and mouse tissue.

Masses observed (kDa)	Antibody manufacturer/Publication	Tissue/cells
43-50, 40-48, 5861 kDa	https://resources.rndsystems.com/pdfs/datasheets/af1074.pdf?v=20210505&_ga=2.34404131.365807519.1620224756-778701540.1493053921	placenta tissue , human testis tissue, bEnd.3 mouse endothelioma cell line and SVEC4-10 mouse vascular endothelial cell line
35, 45, 66	https://www.ptglab.com/Products/JAM2-Antibody-12972-1-AP.htm	Human placenta
42	https://www.sinobiological.com/antibodies/jam-2-jam-b-101895-t32?utm_source=biocompare&utm_medium=platform&utm_campaign=antibody	HeLa Whole Cell Lysate, A431 Whole Cell Lysate
35	https://www.mybiosource.com/monoclonal-human-antibody/jam2/206622	K562 cell lysate, LNCaP cell lysate, A549 cell lysate
31, 35, 45	https://www.lsbio.com/antibodies/ihc-plus-jam2-antibody-clone-1g4-elisa-ihc-wb-western-ls-b5473/140022?trid=247	Human placenta
35	https://www.mybiosource.com/polyclonal-human-mouse-rat-antibody/jam2/9418224	HUVEC cell lysate
46	https://www.antibodies-online.com/productsheets/ABIN3178146.pdf	
39, 28	https://www.thermofisher.com/antibody/product/PA5-30788.html?CID=AFLBC-PA5-30788	human tissue extract
36	http://www.affbiotech.com/goods-6631.html	
36, 45	https://www.epigentek.com/catalog/jam2-polyclonal-antibody-p-74447.html#gallery2	A-549, SH-SY5Y
45, 55, 64-68, 88, 105	https://www.epigentek.com/catalog/jam2-polyclonal-antibody-p-74447.html#gallery2	Hela
31, 45, 70, 88, 99	https://www.epigentek.com/catalog/jam2-polyclonal-antibody-p-74447.html#gallery2	Mouse brain
28-30, 31-35, 45, 70, 99-100	https://www.epigentek.com/catalog/jam2-polyclonal-antibody-p-74447.html#gallery2	Mouse Heart
45, 55	https://www.epigentek.com/catalog/jam2-polyclonal-antibody-p-74447.html#gallery2	Mouse Lung
28, 31, 36, 45, 55, 65, >250	Cunningham et al, 2000, Figure 4	CHO and HSB cells
